# Interventions to Improve the Response of Professionals to Children Exposed to Domestic Violence and Abuse: A Systematic Review

**DOI:** 10.1002/car.2385

**Published:** 2015-06-29

**Authors:** William Turner, Marianne Hester, Jonathan Broad, Eszter Szilassy, Gene Feder, Jessica Drinkwater, Adam Firth, Nicky Stanley

**Affiliations:** ^1^ School for Policy StudiesUniversity of BristolUK; ^2^ Centre for Academic Primary CareUniversity of BristolUK; ^3^ Leeds Institute of Health SciencesUniversity of LeedsUK; ^4^ Public Health and Primary CareUniversity of ManchesterUK; ^5^ School of Social WorkUniversity of Central LancashirePrestonUK

**Keywords:** domestic violence, child safeguarding, systematic review, professional assessment

## Abstract

Exposure of children to domestic violence and abuse (DVA) is a form of child maltreatment with short‐ and long‐term behavioural and mental health impact. Health care professionals are generally uncertain about how to respond to domestic violence and are particularly unclear about best practice with regards to children's exposure and their role in a multiagency response. In this systematic review, we report educational and structural or whole‐system interventions that aim to improve professionals' understanding of, and response to, DVA survivors and their children. We searched 22 bibliographic databases and contacted topic experts for studies reporting quantitative outcomes for any type of intervention aiming to improve professional responses to disclosure of DVA with child involvement. We included interventions for physicians, nurses, social workers and teachers. Twenty‐one studies met the inclusion criteria: three randomised controlled trials (RCTs), 18 pre‐post intervention surveys. There were 18 training and three system‐level interventions. Training interventions generally had positive effects on participants' knowledge, attitudes towards DVA and clinical competence. The results from the RCTs were consistent with the before‐after surveys. Results from system‐level interventions aimed to change organisational practice and inter‐organisational collaboration demonstrates the benefit of coordinating system change in child welfare agencies with primary health care and other organisations. Implications for policy and research are discussed. © 2015 The Authors. *Child Abuse Review* published by John Wiley & Sons Ltd.

‘We searched 22 bibliographic databases and contacted topic experts’

**Key Practitioner Messages:**

- We reviewed published evidence on interventions aimed at improving professionals' practice with domestic violence survivors and their children.
- Training programmes were found to improve participants' knowledge, attitudes and clinical competence up to a year after delivery.
- Key elements of successful training include interactive discussion, booster sessions and involving specialist domestic violence practitioners.
- Whole‐system approaches aiming to promote coordination and collaboration across agencies appear promising but require funding and high levels of commitment from partners.

‘Training programmes were found to improve participants' knowledge, attitudes and clinical competence up to a year after delivery’

## Rationale

Despite the major public health and clinical impact of domestic violence and abuse (DVA), the response of health care professionals to women experiencing domestic abuse is poorly informed and often inappropriate, reflecting its virtual absence in undergraduate education, low profile in postgraduate education and inconsistent presence in continuing professional development (Taskforce on the Health Aspects of Domestic Violence, 2010). Within primary care, the majority of survivors/victims are not identified by clinicians (Feder *et al*., 2011)

‘Within primary care, the majority of survivors/victims are not identified by clinicians’

Over recent years, there have been significant policy developments following high‐profile failures in child protection procedures (HM Government, 2013; Munro, 2011). The impact of changes such as these has been to sensitise health care professionals to the need to ensure that child safeguarding is considered in a systematic and robust fashion. Despite the association of DVA with other types of child maltreatment and the effects of exposure to DVA on the development, educational attainment and mental health of children (Gilbert *et al*., 2009), the issue is not sufficiently addressed in current child safeguarding training, although its relationship to child maltreatment is recognised in national guidance (Royal College of General Practitioners (RCGP) and NSPCC, 2011). The UK General Medical Council (2012) guidance highlights the uncertainty among general practitioners in relation to their child safeguarding responsibilities in the context of DVA, including assessment, mandatory reporting, information sharing and ongoing support to the family. A key recommendation of the UK National Institute for Health and Care Excellence (NICE) DVA guidelines is that health care professionals receive training to identify and, where necessary, refer to children's services children affected by DVA (NICE, 2014).

‘A key recommendation… is that health care professionals receive training’

DVA and child safeguarding are related issues because of the damaging effects on children of being exposed to domestic violence, the overlap between child maltreatment and DVA, and the negative effects on parenting (Hester *et al*., 2007; Stanley, 2011). Yet policy has largely developed on separate ‘planets’ (Hester, 2011). This is particularly striking within the health sector where CS and DVA are often separate components of policy and, in the context of training for a number of health professionals, are insufficiently discussed together.

Although there is considerable research evidence associating DVA and child safeguarding, we need mechanisms for linking them in policy and practice in health care settings.

## Objectives

To identify the types of interventions and their impact on (a) professionals' understanding and responding to both women and their children upon disclosure of domestic violence or of disclosure of child maltreatment in the context of DVA, and (b) improving professionals' assessment of and responses to DVA disclosure.

## Methods

Our methodological approach was guided by the criteria specified in the *Cochrane Handbook for Systematic Reviews of Interventions* (Higgins and Green, 2011). The protocol for this review is registered with the PROSPERO database of systematic reviews (http://www.crd.york.ac.uk/prospero; registration number CRD42013004672).

‘Our methodological approach was guided by the criteria specified in the *Cochrane Handbook for Systematic Reviews of Interventions*’

Searches (with no language restrictions) were made of the international literature for peer‐reviewed and non‐peer‐reviewed studies. As the aim of the review was to be as inclusive as possible, we did not apply any restrictions in the type of study designs considered for inclusion. We considered any type of intervention or significant change in the national or local policy/practice intended to facilitate and improve professionals' response to disclosure of DVA with child involvement and improve professionals' response to child maltreatment in the context of domestic violence. Full details of the methods, details of databases searched and approaches to data synthesis and critical appraisal of included studies can be found in Appendix 1 (see Supplementary Information).

## Results

### Results of the Search

The number of records at each stage of the review is shown in the Preferred Reporting Items for Systematic Reviews and Meta‐Analyses (PRISMA) flow diagram (Appendix 1, Figure 1). The majority of the hits (n = 7552) produced through the electronic searches were deemed ineligible at the first screening stage. Fifty‐one of the 76 papers that were potentially eligible were excluded leaving 21 studies reported in 23 papers.

### Included Studies

Eighteen studies tested individual‐level and three tested system‐level interventions.

#### Individual‐level Interventions

Details of individual‐level interventions are given in Table 1.

**Table 1:** Characteristics and outcomes in studies with individual‐level interventions

Study location	Study design Sample	Intervention	Main outcome results
Berger *et al*. (2002)	Pre‐/post‐test survey design	Initial session a 30‐minute didactic session	1. Attitudes and beliefs about DV: There was a high rate of correct responses to the knowledge questions in all groups at baseline. As a result the only knowledge‐based question in the post‐intervention was related to mandated reporting. After the training, there was no overall change in the number of correct responses to this question
Children's Hospital of Pittsburgh, PA, USA	Trainees (n = 57; 51 paediatric and 6 medicine‐paediatric residents) Staff (n = 27; 5 registered nurse practitioners and 22 faculty)	3 ‐months after the initial session, a 90‐minute teaching session (15‐minute didactic, 12‐minute videotape testimony from DV victims and a 45‐minute role‐play session)	2. Change in the frequency of routine screening practices improved after the intervention. Seventy‐nine participants (96%) believed that screening for the presence of DV was part of their role as paediatric HCP. At baseline, 17 (21%) of the 82 participants reported that they were routinely screening for signs of IPV during well‐child care visits compared with 39 (46%) after attending the education programme (*p* = 0.005). Among participants who attended both educational sessions 25% (9/36) were routinely screening for DV prior to the intervention compared with 46% (16/35) after the intervention (*p* = 0.008) At baseline, 33 (40%) of the 82 participants had identified at least one case of DV in the prior 6 months compared to 45 (53%) after training. Prior to training, 18 participants (22%) were aware of resources for DV victims compared with 45 (53%) after training (*p* < 0.001)
Boursnell and Prosser (2010)	Pre‐/post‐test survey design	Collaborative project (quality improvement study)	1. Awareness of DV policy: Nurses' self‐reported awareness of the policy relating to DV increased significantly after they had completed the training programme
Emergency Department (ED), New South Wales, Australia	Most ED participants (n = 49) were registered nurses (84% pre, 86% first post‐test, 89% second post‐test). The others were enrolled nurses or student nurses	The first phase involved the development of clear guidelines and frameworks acceptable to both ED nursing staff and those from the Violence, Abuse and Neglect Prevention team. This was followed by the development of a flowchart, designated as a ‘pathway’ for use in the identification of DV in ED. The further phases of the project involved the training programme, focus groups to assess the on‐going usefulness of the project and finally a series of file audits which sought to also assess improvements in practice	2. Awareness of responsibilities to DV: Prior to training, approximately half of the nurses (52%, n = 25) said that they were not aware of their responsibilities in DV cases. When they completed the post‐training surveys, this had decreased to only one staff member continuing to report lack of awareness of these responsibilities
		The training programme involved instruction on how to identify three key actions in the pathway for DV presentations in the ED	3. Responding to DV indicators of DV: One month after training, fewer nurses (18%, n = 4) reported that they did not feel confident whilst most (82%, n = 18) of their nurses reported that they felt that their ability to identify DV had increased. The same finding occurred at the 6‐month follow‐up with most nurses (74%, n = 14) reporting that they still felt confident about their improvement in practice due to the project, with only a few of the nurses (25%, n = 5) reporting that did not feel that their improvement in practice had been maintained
			4. Knowledge about referral: After training the number of nurses who reported lack of ability to refer was reduced considerably (27%, n = 6) and remained relatively steady 6 months afterwards (32%, n = 13) Following training, the percentage of participants that reported being able to identify and respond appropriately increased from 48% (n = 24) to 82% (n = 18). This change was sustained at the second follow‐up occasion where a similar number (84%, n = 16) reported believing themselves able to respond appropriately.
			
Centers for Disease Control and Prevention (CDC (2000))	Pre‐/post‐test survey design	The Pediatric Family Violence Awareness Project, a training project for maternal and child HCPs, promoted prevention of and intervention for IPV	Patient's screening status: Each patient's final screening status (ever or never screened) was based on combined data from each phase and was evaluated relative to patient demographics and visit characteristics by two separate logistic regression models
Boston, Massachusetts, USA	Child health care providers (HCPs) n = 14 HCPs, 642 patients and 1352 patient visits	Phase 1 followed a 2‐hour group training session to teach HCPs to implement a brief screening protocol of female patients and mothers of paediatric patients aged 0–12 years during routine visits using a recommended screening schedule	Eleven (79%) of 14 HCPs did not demonstrate increased screening during phase 2, following on‐site services implementation
		Phase 2 followed implementation of on‐site victim services that offered weekly support groups separately for battered women and children using the identical protocol as in phase 1. Between the end of phase 1 and the beginning of phase 2, there was a 3‐month period	Unadjusted individual HCP screening rates varied during both phases from 1.8% to 92.8% during phase 1 and from 0% to 94.9% during phase 2. The degree of change in HCP screening rates also varied widely
Coonrod *et al*. (2000)	Randomised control trial Participants were randomised prior to recruitment (using a computer and stratifying by sex and specialty)	Experimental group:	1. Self‐reported diagnosis of a case of DV sometime between the intervention and the follow‐up (9–12 months after the intervention) Seventy‐one per cent of the residents in the experimental group diagnosed as DV; 52% in the control group did so (RR, 1.35; 95% CI 0.96–1.90; *p* = 0.07) in the 9 to 12 months post‐intervention. Rates of diagnosis differed by specialty (*p* < 0.01)
Maricopa Medical Centre, Phoenix, Arizona, USA	Maryland, medical residents entering in 1995 and 1996	1995: A 20‐minute video presentation *Domestic Violence: More Prevalent Than You Think*, emphasising the importance of screening for DV	2. Change in knowledge on DV; There was a significant effect (*p* < 0.002) of group on post‐intervention: 11 residents in the control group scored a mean percentage correct of 56%; in the experimental group (n = 12) the mean percentage correct was 73%
	Experimental group: n = 53/68 randomised Control group: n = 49/68 randomised	1996: A 20‐minute programme comprising a 9‐minute videotape, *Domestic Violence: The Bottom Line* and a role‐play demonstrating interview techniques for detecting DV	
Cross and Cerulli (2007)	Post‐test survey design	The conference, entitled ‘Understanding Children Exposed to Community Violence: A Conference for Attorneys Committed to Children’, was a typical single‐day professional development training. The conference featured four local speakers who provided information on community violence, local community statistics, evidence‐based research on the impact of violence on children, and the rationale and specific strategies for interviewing children as part of the law guardian role. The main goal was to increase law guardians' knowledge about community and DV and to assist them in identifying and providing appropriate service for child clients	Attendees and a comparison group of non‐attendees also completed a 15‐item questionnaire that was based on a validated instrument designed to test trainees' knowledge, attitudes/efficacy, beliefs and intended practice behaviours following training on IPV (Short *et al*., Unpublished manuscript)
Mid‐size city in upstate New York, USA	Law Guardian Program attorneys		
	Conference group (n = 41) Comparisongroup (n = 28)	Each participant was provided additional reference materials including articles on the topics and copies of the presentations	Results showed that the comparison and conference groups were not significantly different on any demographic variable. T‐tests were conducted to test differences on knowledge, efficacy and practice behaviours for the two groups. Results showed that the groups differed on two of the three variables. Conference participants had statistically significantly higher scores on efficacy and practice behaviours. There was a trend for the conference group to have higher knowledge scores
			
Dubowitz *et al*. (2011)	Cluster randomised trial	The SEEK Model	1. The Health Professional Questionnaire (HPQ): Comparing baseline scores with 6‐, 18‐ and 36‐month follow‐up data, the HPQ
Maryland, Baltimore, USA	18 private practices stratified for size (small, medium, large) Practices ranged from solo to 1 with 32 HPs	Experimental group: HP training The focus was on the significance of targeted problems (parental depression, major stress, substance abuse and IPV) for children's health, development and safety, and how to briefly assess identified problems, including principles of motivational interviewing	revealed significant (*p* < .05) improvement in the SEEK group compared with controls on addressing depression (6 months), substance abuse (18 months), IPV 6 and 18 months) and stress (6, 18 and 36 months), and in their comfort level and perceived competence (both at 6, 18 and 36 months)
	Experimental group: 7 practices, median practice size = 5, range = 1–32. 56 participants	The Parent Screening Questionnaire	2. Review of children's medical records: Before the study, SEEK and control HPs rarely screened for the problems. By medical record data, SEEK HPs improved by >20 percentage points in screening for each risk factor. Controls barely changed
	Control group: 11 practices, median practice size = 3, range = 1–12. 46 participants	A 20‐item yes/no screen for the targeted psychosocial risk factors. It was to be given to ALL parents bringing their child (0‐5) for acheck‐up ‐ its completion was optional	3. Observation of HPs conducting Child Health Supervision Visits: SEEK HP screened for targeted problems more often than did controls based on observations 24 months after the initial training and the medical records (*p* < .001)
		Parent hand‐outs	
		Customised parent hand‐outs for each practice (i.e. local resources) and a web‐based directory of community resources Social worker: A project social worker spent a half‐ or full‐day per week in each SEEK practice. She was available by telephone to HPs and parents during the regular work week	
Feigelman *et al*. (2011)	Randomised control trial	The SEEK Model (for description see above)	1. The Physician Questionnaire on residents' knowledge, attitudes, comfort level, perceived competence and practice Intervention group residents improved more than control subjects on three psychosocial problem scales: Depression, IPV and Stress. This improvement was sustained over 18 months (*p* < 0.01, *p* < 0.03, *p* < 0.04, respectively)
Primary care continuity clinics of a medium‐sized inner‐city paediatric practice, Maryland, Baltimore, USA	Categorical paediatric and combined medicine‐paediatric residents who provided care in continuity clinics		2. Children's medical records were reviewed toward the end of the study to determine physician practice in addressing the risk factors. After the training, intervention residents were far more likely than control subjects to screen parents for the targeted risk factors
	Experimental group: 50 participants		3. Parents' satisfaction regarding doctor‐parent interaction. Parents of children seen by intervention doctors were more satisfied with their child's doctor compared to those seen by control doctors (17.4 vs. 16.9; *p* < 0.01). Group differences were not found, however, at the 6‐month evaluation
	Control group: 45 participants		
Haas *et al*. (2011)	Post‐test survey design	The curriculum was designed to provide participants with an understanding of the dynamics of DV, the process that follows a report of child abuse and/or neglect, and the impact on families when these problems co‐occur. Participants were also exposed to the guiding principles of the three main systems (i.e. child protective services, DV services and courts) as well as law enforcement. Each system was described in terms of their respective roles and responsibilities, risk assessment and safety planning	1. Knowledge (on extent of understanding the legal and/or procedural roles and responsibilities of DV advocates, law enforcement officers and court personnel). The findings indicate that the training did not result in statistically significant changes in the mean levels of these measures. However, there is evidence that the training resulted in some improvements and that these changes varied across DV advocates, law enforcement personnel and court representatives
West Virginia, USA	CPS workers(n = 146 total)	The curriculum was to be delivered by a multidisciplinary training team – to a multidisciplinary audience – of DV advocates, child protective service workers, law enforcement officers and court representatives. A series of 10 regional cross‐disciplinary workshops were conducted throughout the state	2. Attitudes toward collaboration with their interagency partners; whether participants had a positive or negative view of their collaborations with each of the three groups over the past 6 months. Knowledge of the legal roles and responsibilities of other co‐occurrence partners and attitudes based on prior collaborations were shown to be more favourable in the post‐training sample in most cases as there were statistically significant correlations with CPS workers' self‐reported levels of collaboration. Both composite measures between the knowledge and attitudes of CPS workers and levels of collaboration were statistically significant in the comparison and post‐training sample
	Survey respondents represented two samples		3. Perception of the presence or absence of barriers to collaboration. A greater proportion of CPS workers viewed barriers to be related to system‐level factors. Such system‐level factors as high turnover rates, time constraints and too few staff were perceived to be important barriers prior to and after the training. Over 60% of CPS workers in both groups reported these to be important barriers to collaboration. On the contrary, 40% or fewer of respondents viewed individual‐level barriers to be important in curtailing collaboration
			There were significant reductions in the perception of some barriers among CPS workers. An examination of the mean scores showed statistically significant declines for ‘too few staff’ (t = 3.011; *p* = 0.003) and ‘lack of contact between agencies’ (t = 2.617; *p* = 0.010) as perceived barriers to collaboration. Likewise, the perception of ‘lack of interpersonal relationships’ as an obstacle to collaborative efforts was lower in the post‐training sample (t = 3.720; *p* = 0.000). The ‘lack of confidence in counterpart knowledge’ was significantly lower in the post‐training group (t = 2.820; *p* = 0.006)
	1^st^ sample (n = 75) completed the survey on‐site prior to the start of the first training module (comparison group)		
	2^nd^ sample (n = 71) received survey six months after attending training (training/treatment group)		
Johnson *et al*. (2009)Freestanding tertiary care midwestern children's hospital, USA	Pre‐/post‐test survey design (with 3‐month follow‐up)Registered nurses (n = 68 total, female = 65, male = 3)	The 30‐minute educational curriculum for IPV screening As part of the educational session, nurses in groups of two or more viewed a 20‐minute hospital‐produced video about IPV, read through a scripted role‐play and had a discussion	Factor analysis was performed on the baseline Self‐efficacy for Screening for IPV Questionnaire using varimax rotation. Five factors were identified: conflict, fear of offending parent, self‐confidence, appropriateness and attitude. Only fear of offending parent was significantly different from times 1 to 3, indicating that nurses were less fearful after the training. Nurses reported significant improvement (baseline to 3‐month follow‐up) in several self‐efficacy items
			
Knapp *et al*. (2006)	Pre‐/post‐test survey design (with 6‐month follow‐up)	An instructional programme called It's Time to Ask to aid in the identification and intervention for IPV in the paediatric acute care setting	1. Attitudes and beliefs: Participants had consistent, positive changes in attitudes after training that persisted at the 6‐month follow‐up for five items on the questionnaire. Attitudes that did not change showed baseline means already in disagreement with questionnaire statements
Children's Mercy Hospitals and Clinics, Kansas City, MO, USA	Paediatric ED staff (physician, nurses, and social workers) n = 79	The 2‐hour course consisted of three modules and included an evaluation component. First module: basic definitions and concepts regarding IPV in the paediatric health care setting. Second module: addressed attitudes, beliefs and behaviours identified as barriers to screening and intervention. Third module: presented a model protocol for use in the paediatric acute care setting	2. Self‐efficacy: Participants reported significant, positive changes for all seven self‐efficacy statements at one or both of the post‐training evaluations
			3. Behaviours/clinical practice: The only changes in behaviour were observed at 6 months
Lelli (2011)	Pre‐/post‐test survey design	Bibliotherapy: reading professional literature and children's literature pertaining to DV	The pre and post survey responses were analysed and coded to determine if preservice teachers' attitudes and views about identifying signs of DV changed after the professional development and readings of the given literature
Catholic liberal arts college in southeastern Pennsylvania, USA	40 undergraduate students from three classes of a reading methods course which is generally taken during senior year prior to student teaching		The results showed an increase in preservice teachers' knowledge and skills pertaining to recognizing signs of DV in behaviours of the students they teach. The data further revealed that the increase was due to the use of children's literature as part of instruction and trade journal articles as a part of teachers' professional development
			
McCauley *et al*. (2003)	Pre‐/post‐test survey design	The video, *ASSERT: A Guide to Child, Elder, Sexual, and Domestic Abuse for Medical Professionals*, was developed by experts from medicine, social work, nursing and law. The video featured role‐plays to demonstrate different approaches to these difficult clinical encounters. The settings for watching the video and completing the questionnaire varied from staff meetings, to in‐services, to CME meetings called specifically to view the video	A questionnaire with 13 knowledge and 12 attitude variables was specifically developed to assess knowledge and attitude changes
Baltimore, Maryland, USA	120 physicians and 172 other personnel (e.g. nurses, social workers) at 24 sites associated with four academic medical centres completed paired questionnaires		120 physicians and 172 other personnel (e.g. nurses, social workers) at 24 sites associated with 4 academic medical centres completed paired questionnaires. There was significant improvement for physicians in 77% of the knowledge items and 75% of the attitude items from pre‐ to post‐viewing questionnaires. A total of 73% of viewers would recommend the video to colleagues
McColgan *et al*. (2010)Urban tertiary care paediatric hospital Philadelphia, PA, USA	Pre‐/post‐test (3‐ and 8‐months) survey designPaediatric residents (n = 52 baseline/ 72 recruited)	The multifaceted IPV intervention consisted of the following: IPV screening and intervention protocol. Paediatric residents were trained to screen all‐female caregivers for IPV according to the Family Violence Prevention Fund consensus statement Establishment of on an on‐site IPV counsellor: available on‐site Monday through Thursday (continuity clinic days) and via pager on Friday Resident ‘champions’ in each continuity clinic. Responsibilities of the resident ‘champions’ included: preparing and presenting a 25‐minute talk for their clinic team about ‘IPV screening in the paediatric setting’, encouraging IPV screening and obtaining monthly feedback from fellow residents about barriers to screening for IPV IPV training for the social work staff, attending physicians and resident ‘champions’. Five of the eight APC attending physicians and the four resident “champions” attended a 2‐hour training session on IPV screening in the paediatric setting. The medical Social Work Department received 5 hour of IPV training Training of paediatric residents. Consisted of the following: a 1‐ hour ‘grand rounds’ presentation to the medical staff; two 1‐hour ‘noon‐conference’ talks on IPV for the residents; and one 25 minute ‘pre‐clinic talk’ on IPV screening presented by the resident ‘champion’ of that continuity clinic team	Programme efficacy was evaluated through (1) resident surveys and (2) chart reviews 1. Resident questionnaire assessing their perceived knowledge, comfort, attitudes, barriers and screening practices regarding IPVChanges in attitudes, and perceived knowledge of IPV. Compared to baseline, the 3‐month follow‐up survey revealed significant improvements in perceived knowledge of appropriate IPV screening questions (47.1% vs. 100%), referral sources (34.3% vs. 82.9%), and the relationship between child abuse and IPV (52.9% vs. 97.1%)
			Changes in barriers to IPV screening. ‘Knowledge of how to screen’ and ‘not knowing where to refer positive screens’ did not appear as barriers in the 3‐month follow‐up survey
			2. Chart review: Documentation of IPV screening and referrals to IPV counsellor Chart review: Changes to documentation of IVP screening. Significant and sustained improvements in documentation of IPV screening were noted. IPV screening rates improved from 0.9% at baseline to 36% at 3 months, and remained elevated to 33% at 8 months
			Chart review: IPV counsellor's charts. The IPV counsellor had received 107 referrals for IPV during the first 12 months of the intervention: 50 during the first 6 months and 57 during the next 6 months. Of these, 25 (23%) were from the ambulatory paediatrics clinic and 77% were from other hospital departments
Mills and Yoshihama (2002)	Pre‐/post‐test survey design	Two types of training were developed and offered in 1995: The One‐Day Programme consisted of didactic teaching and a role‐play exercise that encouraged CSWs to test their new skills in a practice interview with a battered woman The Fellow's Programme consisted of six monthly one‐day workshops and was designed to provide in‐depth and leadership training for a selective group of CSWs and supervisors	At post‐test, the participants in the One‐Day Programme were significantly less tolerant of DV (t = −5.44, *p* < .001) and more likely to view DV as a social problem (t = 2.32, 12 < .001). They were more likely at post‐test to consider assessing whether the mother is being abused as one of the first tasks of a CSW (t = 3.93, *p* < .001) The likelihood of referring the mother and father to couples counselling decreased slightly following the training but not significantly. Participants were significantly less likely to view battered women as incapable of protecting children and more likely to view women staying in abusive relationships due to their fear of losing custody of the children (t = −3.92, *p* < .001; t = 7.66, 12 < .001, respectively). Participants perceived themselves significantly more competent to respond to DV cases following the training (t = 10.21, *p* < .001)
Orange County, Los Angeles, USA	Children's services workers (CSWs) n = 179		
			
Prather (2003)	Post‐test survey design (study 2)	Study 2 was designed to use the Knowledge and Attitudes Questionnaire (KAQ), Scenario Responses (SR) and student Journal Responses (JR) to evaluate the impact of the Child Abuse and Family Violence Course (CAFVC) on these obstacles	Study 2 measures:
USA	Post‐test scores of participants on the KAQ were compared with a matched control group selected from participants of Study 1		1. The KAQ developed and empirically evaluated in study 1 and used to examine changes in participants' avoidant reactions, prejudicial attitudes, attitudes about oppression and recognition of abuse Results indicate the CAFVC was effective in reducing the barriers of limited knowledge, avoidant reactions, beliefs about role and sexist attitudes in the context of abuse
	Participants were recruited from members in the National Association of School Psychologists, attendees at the British Columbia/Washington Association of School psychologists' annual conference and students from training institutions that prepare school psychologists Study 1: n = 186 Study 2: n = 23 students	The child abuse and family violence course (CAFVC) included specific content and pedagogy in order to directly address the barriers that keep professionals from effectively responding to child abuse and family violence. The course was taught at a large public university in the fall quarter of 2000. Course length was 10‐weeks, with class meeting for 2 hours of instruction once a week. The interdisciplinary guest speakers were used to provide information to participants as well as to allow students to form collaborative working relationships with them	2. SR. Participants were presented with a hypothetical case and asked how they would respond. The same case scenario was used in the pre‐ and post‐tests. Paired‐sample t‐tests of participants' SR scores indicated the CAFVC increased the application of effective interventions3. JR. Participants were also asked to keep a weekly journal as a reflection of their thoughts, feelings and concerns in response to the CAFVC (to provide a means to evaluate whether there were negative effects of the course on participants and to examine affective reactions and avoidant behaviours). JR qualitative analyses indicated a three‐stage pattern of emotional activation that resulted in decreased avoidance
			
Saunders *et al*. (2006)USA	Two types of evaluation designs were used: post‐test only and pre‐and post‐test	The state's DV training unit conducted the training and encouraged welfare managers and workers to attend a 1‐day training session aimed at helping them to identify and understand DV, develop safety plans and make referrals. It specifically covered several key issues: the definition and nature of DV, ways victims try to protect themselves and their children, guidelines for interviewing clients, initial interview questions, identifying DV, lethality indicators, helpful interventions and safety planning tools. A highly experienced DV specialist conducted 63 trainings at 10 sites throughout the state	Two vignettes were constructed for the study: one with obvious content about DV and one with DV risk markers but no mention of DV. Including this second vignette helped with the assessment of the ability to detect abuse Participants indicated the likelihood, from 0% to 100%, of their making various responses if they were interviewing the hypothetical client. Items were derived from the goals of the training and current policy
	Post‐test evaluation (n = 192) (May 1998 and October 1998)		Training effects: Trained workers reported a greater likelihood of referring clients to couples counselling, developing a safety plan and reporting to CPS. However, the latter two findings did not hold after controlling for demographic and background variables. The difference on the safety planning item was not significant after controlling for gender, educational level, years of experience in social services and prior information obtained about DV. The difference on CPS reporting did not hold after controlling for years of experience. There were no significant differences on any of the other items
	Pre‐/post‐test evaluation (n = 67) (July 2000 and December 2000)		
			
Shefet *et al*. (2007)	Pre‐/post‐test (6‐month) survey design	The programme included three branches: IPV, child abuse and elder abuse. All branches shared common educational goals and differed in unique emphases related to each. Each branch developed an 8‐hour workshop, based on SPs. Each workshop was developed by a national committee of DV experts and included eight scenarios reflecting common DV‐related encounters with patients and/or family members and care takers. Each physician encountered two scenarios and actively viewed, via a one‐way mirror, four others. All encounters were audio‐visually recorded. Encounters lasted 12 minutes each, after which 4 minutes were allotted to documentation and comments, and another 4 minutes for a private, undocumented oral feedback by the actor. At two points during the workshop—halfway through and at the end—the participants assembled in a debriefing room and viewed selected segments of recordings from each encounter. Key points from each of the scenarios (content and/or communication skills) were discussed under the instruction of both a physician and a social worker specialising in DV	1. Perceived capabilities Perceived capability in diagnostic skills, communication skills, knowledge of favourable intervention, graded on a scale of 1 (not at all capable) to 4 (capable to a large extent), had increased by 0.29 to 0.6. All increments were statistically significant (*p* < 0.05)
Israel Center of Medical Simulation, Israel	Physicians (including general practitioners, residents and specialists in relevant primary care fields, from both outpatient and inpatient settings)		2. Reported case management Frequency of routine screening of DV (on a scale of 1 = always to 4 = never) has increased (mean score decreased by 0.19, *p* < 0.03). Reported actions: Participants were given a list of nine actions, and were asked how often, upon encountering a case suspicious of DV, they take these actions (on a scale of 1 = never, to 4 = always) All frequencies of reported actions taken were increased, including documentation of the violence in the medical chart, empowering the patient, providing the patient with relevant information and referring him/her to relevant agencies for treatment. All but one increment were of statistical significance
	Pre‐/post‐test (n = 74) (recruited n = 141)		3. Perceived intervention barriers At follow‐up, lack of knowledge and lack of communication skills, as well as unfamiliarity with support systems (‘I don't know where to refer’) and psychological difficulties (‘I am afraid it will find it difficult to cope emotionally’) all received significantly lower scores, which indicates an improvement in the physicians' attitudes regarding these barriers
Young *et al*. (2008)	Pre‐/post‐test survey design	The Helping Child Victims of Domestic Violence: Implications for School Personnel training included information about the dynamics of DV, the effects on children, interventions and community resources The training was presented by personnel from the Rural Justice Institute (RJI), a research and service entity of Alfred University. Primary presenters included a doctoral‐level school psychologist and an educational specialist. When scheduling permitted, the local DV service provider also participated in the training by presenting the programme introduction (dynamics of DV) or providing the audience with a presentation of their services at the end of the training The workshop training was approximately an hour and a half in length	Scores in knowledge and attitudes regarding DV (12 statements at pre‐test, 11 items at post‐test)
Four counties (18 different locations) in rural western New York, USA	644 school personnel		Overall results were favourable, with 10 out of 11 questions showed significant improvement (*p* < 0.001) from pre‐test to post‐test.

HCP = Healthcare providers; ED = Emergency Department; HPs = Child health care professionals; CPS = Child protective service; CME = continuing medical education; APC = Ambulatory Pediatrics Clinic; SPs = standardized patients; IPV = intimate partner violence; CI = Confidence intervals; RR = Risk ratio; DV = domestic violence.

A total of 2018 participants were included in the 18 studies, the majority being clinicians. Three of the 18 studies were randomised controlled trials (RCTs) (one a cluster RCT), 12 studies utilised a pre‐/post‐test survey design and three used a post‐test only design. The majority of the studies were conducted in paediatric settings in the USA.

‘A total of 2018 participants were included in the 18 studies, the majority being clinicians’

Individual‐level interventions were all educational or had an educational component; they all focused on promoting prevention of(IPV) (not violence by other family members) by targeting participants' attitudes towards IPV and knowledge of its detrimental effects on victims and their children, followed by practical measures that professionals could take. Appendix 2 summarises details of the training programmes in the included studies (see Supplementary Information).

Eight discrete training programmes were identified. In two studies, the interventions were multifaceted (e.g. the SEEK Model of paediatric care (Dubowitz *et al*., 2011; Feigelman *et al*., 2011)). Contents of, or topics covered in, the training programmes were not consistently reported in the majority of studies. Teaching methods were also not clearly reported. We could discern that teaching methods were either exclusively didactic (e.g. Berger *et al*., 2002; Cross and Cerulli, 2007) or instructional (e.g. Boursnell and Prosser, 2010).

Programme delivery formats were reported in the majority of studies; these included group presentation, small‐group training, film and video and bibliotherapy. The duration of the training intervention programmes in the included studies varied; six interventions were brief and seven were longer, lasting from 90 minutes to one day (8 hours). Booster training sessions (lasting between 1 hour and 90 minutes) were included in three studies (Dubowitz *et al*., 2011; Feigelman *et al*., 2011; Berger *et al*., 2002).

### System‐level Interventions

System‐level interventions aimed to effect changes in organisational practice (Wills *et al*., 2008) and inter‐organisational collaboration between child welfare and DVA service providers (Banks *et al*., 2008b) to implement strategies in the prevention of DVA (Shye *et al*., 2004) (see Table 2). All system‐level intervention studies utilised a pre‐/post‐test survey design. With the exception of the New Zealand study (Wills *et al*., 2008), they were all conducted in the USA.

‘System‐level interventions aimed to effect changes in organisational practice’

‘Training programmes were found to improve participants' knowledge, attitudes and clinical competence up to a year after delivery’

**Table 2:** **C**haracteristics and outcomes in studies with system‐level interventions

Study location	Study design Sample	Intervention	Main outcome results
Banks *et al*. (2008b) USA	Pre‐/post‐test survey design	The *Greenbook* Demonstration Initiative	Surveys of child welfare caseworkers showed significant changes in several areas of agency policy and practice, including regular domestic violence training, written guidelines for reporting domestic violence, and working closely and sharing resources with local domestic violence service providers
Main reference	Five sites Direct service workers N = 578 (total)	A multisite developmental evaluation of six demonstration sites that received federal funding to implement *Greenbook* principles and recommendations over a 5‐year demonstration period	Case file reviews show significant increases in the level of active screening for domestic violence, although this increase peaked at the midpoint of the initiative
	Cases reviewed (across sites) Time 1: 616 Time 2: 642 Time 3: 562	*Greenbook* principles for guiding reforms in child welfare systems include establishing collaborative relationships with domestic violence service providers and dependency courts; assuming leadership to provide services and resources to ensure family safety for those experiencing child maltreatment and adult domestic violence; developing service plans and referrals that focus on safety, stability and the wellbeing of all victims of family violence; and holding domestic violence perpetrators accountable (National Council of Juvenile and Family Court Judges, 1999)	These findings, coupled with on‐site interview data, pointed to the importance of coordinating system change activities in child welfare agencies with a number of other collaborative activities
Banks *et al*. (2008a) USA	Pre‐/post‐test survey design	The *Greenbook* demonstration initiative (for description see above). This article examines how the demonstration sites developed collaborations in accordance with the Greenbook foundation principles and associated recommendations, including the following: How did the collaborations organise and plan their work? Did the collaborative bodies reflect the Greenbook guidance? What facilitators and obstacles were most salient to the work? How were they addressed? What activities did the collaborations plan to implement policy and practice change in the three primary systems?	A stakeholder survey aiming to capture the dynamic factors contributing to project planning, activity implementation and the status of the collaboration at each site showed that the measures clustered around three factors: leadership, community context and resources. Stakeholders were most likely to agree that senior managers and directors of key organisations saw the co‐occurrence of DV and child maltreatment as a problem in the community and were least likely to agree that the community already had resources, such as available data, funding, and a high level of expertise and training, invested in the issue of co‐occurring child maltreatment and DV Stakeholder interviews on the process and perceived impact of collaborative work. Comparing responses over time, stakeholders were significantly less likely to agree that existence and accessibility of data were an obstacle. stakeholders were significantly less likely to agree that existence and accessibility of data were an obstacle at follow‐up: lack of resources, burnout of participants, conflicting organisational cultures, lack of leadership buy‐in and lack of accountability The top collaborative facilitators (e.g. involvement, commitment and leadership) did not change much over time, given that the top six rated facilitators at baseline were also the top six at follow‐up. At follow‐up, the relationships among collaborative members and agency staff received the highest ratings by survey respondents. Over time, only one facilitator showed significant changes in agreement. Stakeholders were significantly less likely to agree that the involvement of key agencies and groups was a facilitator at follow‐up
Banks *et al*. (2009)USA	Pre‐/post‐test survey designThree of the demonstration sites as case studies^a^	The *Greenbook* demonstration initiative (for description see above)The purpose of the study was (a) to examine collaborative activities occurring between child welfare agencies and domestic violence service providers and (b) to investigate whether there was a relationship between collaborative efforts and domestic violence policy and practice in child welfare agencies	Findings from the cross‐sectional data revealed that in almost three‐quarters of the communities, formal collaborative activities existed between child welfare and domestic violence agencies. The data did not demonstrate a relationship between these activities and child welfare policy and practice related to domestic violence. Longitudinal case study findings from the Greenbook evaluation did reveal some changes in child welfare policy and practice in association with the implementation of activities that increased collaboration between child welfare and domestic violence service providers. Improvements were found in child welfare agency screening and assessment, advocacy for adult domestic violence victims and multidisciplinary approaches to case planning. The extent to which changes were observed varied across the sites, and appeared to be related to the specific planning approach undertaken in each community
			
Shye *et al*. (2004)USA	Clinicians^b^n = 273Female patients ^c^ n = 1925 and n = 1979 for the pre‐ and post‐intervention	Two implementation strategies Basic Implementation Strategy (BIS). The task force's implementation strategy included writing and disseminating a DV guideline,^d^ traditional continuing medical education^e^ and clinical^f^ and environmental supports and cues to increase clinician inquiry^g^ and patient self‐disclosure^h^ of DV exposure. An article describing the signs and dynamics of DV and encouraging HMO members to discuss DV problems with their primary care clinicians appeared in the HMO's member newsletter. The HMO allotted 4 hours/month to the paediatrician co‐chair of the task force to oversee implementation Augmented Basic Implementation Strategy (ABIS) The ABIS augmented the BIS by giving medical office social workers^i^ paid time, funded by the research project, to assume a structured role as DV social change agents (5.2 months of full‐time employment for the 18‐month study period for all the ABIS social workers together)	The ABIS was associated with significantly greater improvement only on knowledge relating to the pros of routine inquiry (β = 0.32, *p* < 0.0001)
			The ABIS was associated with significantly greater improvement on process of change (b = 0.38, *p* < 0.0001). Post‐intervention scores on perceptions of the medical office social workers as DV experts indicated that improvement was strongly associated with exposure to the social workers' social change agent role in the ABIS arm
			The ABIS had no greater effect on inquiry rates than the BIS rather, inquiry rates were a function of patient characteristics and clinician specialty
Wills *et al*. (2008)New Zealand	Over 700 staff	A formal organisational change approach was used to implement the New Zealand Family Violence Intervention Guidelines in a mid‐sized regional health service. The approach included obtaining senior management support, community collaboration, developing resources to support practice, research, evaluation and training,^j^ ^k^	Referrals. It is reported that the number of notifications from HBDHB to CYFS had increased from 10 per quarter to 70 per quarter. CYFS reports indicated that notifications were appropriate and informative, and that interagency relationships were strengthening
			Screening for partner abuse is also reported to have been increased in most services, with rates between 6% and 100% recorded during the 2005/06 years, although there was considerable variability in the rate of screening between services. The number of women disclosing abuse was also increased, as was the amount of referral information provided

The three sites for the case studies were selected based on a combination of factors: completeness of the data collected, representativeness of the challenges and obstacles encountered by all six demonstration sites, and generalisability to other communities.

Participants included all IM, FP, health appraisal (HAP), paediatric and OB/gyn physicians, physician assistants and nurse practitioners in the HMO's main metropolitan area.

Response rates for the pre‐ and post‐intervention female patient surveys were 85.8% (n = 1652) and 80.7% (n = 1598), respectively.

The guideline adopted a ‘routine inquiry’ rather than a universal screening approach, recommending that primary care clinicians routinely ask about DV exposure of female patients and mothers of paediatric patients at ‘health maintenance visits’ (e.g. visits for no acute care including routine check‐ups, routine pregnancy visits and ‘well‐baby’ care) and of all patients whose symptoms suggest abuse.

The task force organised a half‐day conference to train DV response team members and other clinicians.

The task force charged local medical office managers with setting up DV response teams (consisting usually of nurses, medical assistants, social workers and occasionally a female physician) to intervene with identified DV‐exposed persons.

l Two primary care clinician task force members wrote an article describing the clinician's role in response to DV for the HMO's local medical journal.

Thirty plastic dispensers containing ‘calling cards’ with information about community resources for DV victims were placed in all the HMO's restrooms. Printed materials were developed and distributed, including patient brochures and pocket reminders for clinicians about screening, safety assessment, safety planning and community referral resources.

The social workers' role involved: (1) conveying information to clinicians about DV prevalence and risk markers, dynamics of abusive relationships, etc.; (2) advocating an active primary care clinician role in secondary prevention; (3) elucidating the appropriate goals of screening and intervention activities; and (4) modelling secondary prevention skills (i.e. asking patients about DV, danger assessment, documenting abuse, etc.). They undertook these activities in department meetings and in individual ‘academic detailing’‐style contacts with clinicians.

Formal pre‐post evaluations were conducted of the training identifications of partner abuse.

Training in child and partner abuse is mandatory in services primarily serving women and children. Training occurred only after the other systems (e.g. policy, documentation and supervision) were in place. Adult education principles are applied. Full‐day training is provided including lectures, interactive sessions and modelling and practising risk assessment using role‐play. Staff are taught to routinely include a question about partner abuse in their social history and the ‘dual assessment’ model was taught.

CYFS = the Department of Child, Youth and Family Services; HBDHB = Hawke's Bay District Health Board.

The Greenbook demonstration initiative is reported in three papers (Banks *et al*., 2008b, 2008a, 2009) reporting on the rationale and results of the initiative's multisite evaluation which aimed to put into practice Greenbook principles and recommendations over a five‐year demonstration period. Greenbook principles for guiding reforms in child welfare systems refer to: the establishment of collaborative relationships with DVA agencies and juvenile/dependency courts; assuming leadership to provide services and resources to ensure family safety for those experiencing child maltreatment and adult DVA; developing service plans and referrals that focus on safety, stability and the well‐being of all victims of family violence; and holding domestic violence perpetrators accountable (National Council of Juvenile and Family Court Judges, 1999). Six demonstration sites representing a diverse group of communities that varied in terms of population, culture and geography received US federal agency funding to implement the Greenbook recommendations.

‘Six demonstration sites, received US federal agency funding to implement the Greenbook recommendations’

The study by Wills *et al*. (2008) reports on a formal organisational change approach involving the implementation of the New Zealand Family Violence Intervention Guidelines in a mid‐sized regional health service. The approach included obtaining senior management support, community collaboration and developing resources to support practice, research, evaluation and training. Training in child and partner abuse occurred only after the other systems (e.g. policy, documentation and supervision) were in place. Full‐day training was provided including lectures, interactive sessions and modelling, and practising risk assessment using role‐play. Staff were taught to routinely include a question about partner abuse in their social history and the ‘dual assessment’ model was taught.

Finally, the effectiveness of two system‐level multifaceted quality improvement approaches to enhancing the secondary prevention of domestic violence in primary care settings was compared in the Shye *et al*. (2004) study. Two approaches were tested: Basic Implementation Strategy and Augmented Basic Implementation Strategy. The characteristics of each implementation strategy are summarised in Table 2.

### Outcomes

In this section, we summarise outcome measures in the primary studies. This information is presented in Table 1 (for individual level) and Table 2 (for system‐level interventions).

Measures of knowledge, attitudes, perceived competence and screening practice varied between studies. The measures used in the studies were: (1) questionnaire‐based measures, or (2) vignette‐based measures that used hypothetical scenarios to assess participants' knowledge, attitudes, comfort level, perceived competence and screening practice.

‘Measures of knowledge, attitudes, perceived competence and screening practice varied between studies’

Of the 18 individual‐level studies that utilised knowledge, attitudes, competence and/or screening practice measures, administration of outcome measures varied from 48 hours to more than six months following the training programme. Only two studies reported repeated outcome measures (Dubowitz *et al*., 2011; Feigelman *et al*., 2011). The time span where similar measures were assessed in system‐level interventions ranged from five years (e.g. Banks *et al*., 2008a, 2008b; Wills *et al*., 2008) to one year (Shye *et al*., 2004). Ten studies employed additional behaviour change measures of participants' screening practice and IPV identification rates, but also on referral rates to an IPV counsellor (McColgan *et al*., 2010) or to children's services (Wills *et al*., 2008). No studies measured parental or child outcomes, other than parent satisfaction (Feigelman *et al*., 2011).

### Effects of Interventions: Summary of Findings

Appendices 3a and 3b (see Supplementary Information) report full details of outcome measures results for pre/post, post‐test only studies and RCTs, respectively. Appendix 3c reports outcome measures results for system‐level interventions.

## Knowledge

Knowledge outcome data were reported in both individual‐ and system‐level interventions. In the majority of the pre‐/post, post‐test only studies that set out to examine the effects of training interventions, significant improvement in participants' knowledge scores was reported. Training interventions tested under randomised controlled conditions generally supported this trend. Results from the three system‐level intervention studies also report similar significant increases in participants' knowledge about: (a) resources for DVA, training opportunities and DVA operational guidelines (Banks *et al*., 2008b, 2008a); (b) the pros of routine enquiry about DVA (Shye *et al*., 2004); and (c) with identifying and managing child and partner domestic violence (Wills *et al*., 2008).

‘Knowledge outcome data were reported in both individual‐ and system‐level interventions’

## Attitudes

The majority of the pre‐/post/post‐test only studies reported significant improvements in participants' attitudes towards a number of DVA‐related attitude items. Only the Feigelman *et al*. (2011) RCT reported improvements on this domain, though there were no available data to compute an effect size. Of the system‐level intervention studies, only the Wills *et al*. (2008) study reported positive changes in participants' attitudes, though its magnitude could not be estimated as data were not available.

## Competence

Competence outcome data were reported in both individual‐ and system‐level interventions. In all of the pre‐/post/post‐test only studies that set out to examine the effects of training interventions, significant improvements in participants' self‐perceived competence scores were reported post‐intervention. Results from two RCTs strongly supported this trend though IPV‐specific data were not available. Only one of the system‐level interventions (Wills *et al*., 2008) studies indicated positive changes in participants' competence, though, due to data being unavailable, its magnitude could not be estimated.

‘Competence outcome data were reported in both individual‐ and system‐level interventions’

## Screening Practice

Screening practice data were reported in both individual‐ and system‐level interventions. All of the pre‐/post/post‐test only studies that set out to examine the effects of training interventions reported significant improvements post‐intervention in participants' self‐reported screening practice scores. Results from two RCTs offered mixed results on this outcome with Coonrod *et al*. (2000) reporting a significant effect and Dubowitz *et al*. (2011) detecting no significant effect.

Two system‐level intervention studies provided data for this outcome. In the Banks *et al*. (2008a) study, results from caseworkers' surveys during the different stages of the implementation of the Greenbook initiative did not show any significant changes on a number of clinical practice items; the authors offered the huge variability in responses between sites and the high scores at baseline as possible reasons for this finding. In the Wills *et al*. (2008) study both screening and referral rates were increased.

‘Screening practice data were reported in both individual‐ and system‐level interventions’

## Behaviour Change

The significant improvements in IPV identification/screening practice and referrals are consistent with the positive results of the self‐reported knowledge, attitudes and competence outcome measures reported above. The same pattern was observed for both individual‐ and system‐level intervention studies.

## Parent and Children Outcomes

With the exception of the Feigelman *et al*. (2011) study, outcomes for parental and children's outcomes were not measured in any of the included studies. In that study, patient‐rated clinical interactions were significantly more positive compared to control doctors.

## Discussion

### Implications for Practice

Our overall interpretation is that training programmes aiming to improve the response of professionals to the exposure of children to DVA, of the types described in the individual‐level interventions section of this review, improve participants' knowledge, attitudes and clinical competence up to a year after the intervention. Elements of effective interventions include an added experiential or post‐training discussion component (alongside the didactic component), incorporating ‘booster’ sessions at regular intervals after the end of training, advocating and promoting access to local DVA agencies or other professionals with specific DVA expertise, and finally, drawing from a clear and well‐articulated protocol for intervention.

‘Training programmes … improve participants' knowledge, attitudes and clinical competence up to a year after the intervention’

Our synthesis of primary studies documented multidimensionality in training programmes' contents, methods and delivery. This is an important finding in itself. To date, programmes have been categorised dichotomously as active/passive or behavioural/instructional. Our descriptive analysis shows this categorisation to be over‐simplistic as most programmes that we reviewed were multifaceted with multiple components. Programmes covered multiple topics, used teaching strategies in combination such as discussion, modelling, role‐play, rehearsal and feedback, and integrated active/passive and behavioural/instructional approaches in one session (e.g. a video or DVD presentation and then partake in activities). The contribution to effectiveness of programme content, methods and delivery will require documentation using standardised data collection tools in future studies.

‘Most programmes that we reviewed were multifaceted with multiple components’

There was some evidence that improvements in perceived competence can be translated into changes in clinical practice, as documented by clinical record audits. However, perceived competence gains were not sustained consistently over time, indicating the need for reinforcement (e.g. booster sessions). The consistency of results for similar outcome measures evaluated in the three RCTs strengthens the evidence. On the other hand, in the absence of measures of harm, it is unclear whether these training programmes may also have harmful consequences in the form of parental anxiety and child fear or anxiety. It is also uncertain whether the interventions result in greater odds of disclosures of past or current DVA from mothers and children who have contact with professionals who are the targets of the intervention programmes.

Currently, different professional settings implement a variety of interventions aimed at increasing professionals' understanding of, and response to, both women and their children upon disclosure of DVA or of disclosure of child maltreatment in the context of DVA and improving professionals' assessment of, and responses to, DVA disclosure. These training programmes need to be integrated with a multiprofessional and interagency response (Cameron *et al*., 2000; Sloper, 2004). In this respect, the results of the handful of system‐level intervention studies are encouraging and point to the importance of coordinating system change activities in child welfare agencies with other collaborative activities between primary partner systems and DVA specialist organisations. While promising, the commitment for continuous work by all partners was highlighted as one of the most challenging aspects of collaborative initiatives aiming to deliver an integrated DVA policy and practice and improve outcomes for families. Further studies are needed to identify the optimal operational parameters of such strategies.

‘Training programmes need to be integrated with a multiprofessional and interagency response’

### Strengths and Limitations

In conducting this systematic review, our aim was to reduce bias in synthesising the available evidence of interventions to improve responses of professionals to children exposed to DVA. We have used a pre‐specified, transparent and reproducible methodology including a comprehensive literature search, so it is unlikely that we have missed any studies that fulfil our inclusion criteria, although there have been studies published subsequent to July 2013 that fulfil our inclusion criteria, such as Szilassy *et al*.'s (2013) analysis of knowledge outcomes from interprofessional training in DVA and child safeguarding. Identification of relevant studies was duplicated independently and data extraction was validated. We are not aware of any other systematic reviews aiming to identify the effects of training programme interventions for responses to DVA disclosure or of disclosure of child maltreatment in the context of DVA.

The definition of DVA in the included studies was not clearly reported and we could not identify whether the training programme content included men as both victims and perpetrators. In some studies, there is reference to ‘family violence’ as well as ‘IPV’, which may or may not suggest different sets of individuals and may also not include men as victims. The uncertainty as to whether both women and men were included as victims and/or perpetrators in the included studies puts into question whether the training programmes may or may not be covering the whole picture regarding DVA. While heterosexual women are the largest DVA victim group (Smith *et al*., 2010), male victims and/or perpetrators of DVA may also attend general practice and may disclose DVA (Westmarland *et al*., 2004; Hester, 2009). Initiatives to address this gap have been recently undertaken by the first study of men and DVA in general practice (Hester *et al*., forthcoming). Members of the same research team have piloted a training intervention for general practices on asking men about DVA and responding appropriately (Williamson *et al*., 2014). Limitations of the review were weak study designs of the primary studies and incomplete reporting of interventions and outcomes.

‘We could not identify whether the training programme content included men as both victims and perpetrators’

### Implications for Research

Further evidence is required to assess the effectiveness of training and system change programmes aiming to improve the response of professionals to the exposure of children to DVA. The current evidence on training programmes is primarily focused on improvements in participants' knowledge (both factual and applied knowledge) and practice (screening behaviours) and to a lesser extent on assessing harm (child anxiety or fear) and disclosures of past or current domestic violence. None of the primary studies evaluated online programmes.

There remains uncertainty about whether these training and system change interventions improve outcomes for parents and children. Those outcomes need to be measured in future evaluations of interventions addressing the needs of children exposed to DVA, as do potential moderators of intervention effects in the form of child, programme, and contextual characteristics. Such evidence is a necessary precursor to assessing programmes' cost‐effectiveness.

‘There remains uncertainty about whether these training and system change interventions improve outcomes for parents and children’

Finally, programme evaluation needs to embrace randomised study designs, consistent typologies (and adequate descriptions) of training programmes and common outcome measures which allow studies to be compared and results pooled in meta‐analyses. Other design features that warrant particular attention in future studies include those domains associated with risk of bias: randomisation of study participants, allocation concealment, blinding of outcome assessors, reporting of attrition and analysis based on intention to treat. In conjunction with qualitative studies, these trials will inform the development of policy in one of the most challenging issues that face professionals who care for children.

‘These trials will inform the development of policy in one of the most challenging issues that face professionals who care for children’

## Funding

This report is independent research commissioned and funded by the Department of Health Policy Research Programme (Bridging the Knowledge and Practice Gap between Domestic Violence and Child Safeguarding: Developing Policy and Training or General Practice, 115/0003). The views expressed in this publication are those of the author(s) and not necessarily those of the Department of Health.

## Supporting information

Supporting info item
