# Supplementary material for: Interventions to Improve the Response of Professionals to Children Exposed to Domestic Violence and Abuse: A Systematic Review
Source: Child Abuse Rev. 2015 Jun 29;26(1):19–39. doi: 10.1002/car.2385 (PMC5363379; doi:10.1002/car.2385)
Supplement: Supplementary file 1 — Supporting info item [file CAR-26-19-s001.zip › CAR-071-14-SI-RESPONDS---Appendix-3a-NEW--results-for-pre-post-test-studies--individual-level-study--March2015.docx]

**Appendix 3a: Results in pre-/post-test, post-test only studies (individual-level interventions)**

| **Study** | **Knowledge measures** | **Attitudes measures** | **Self-efficacy /competence measures** | **Practice Behaviours/clinical (screening) practice** | **Behaviour change** | **Harm**  **Parental or child anxiety or fear** |
| --- | --- | --- | --- | --- | --- | --- |
| **Berger *et al.* (2002).** | The only knowledge-based question in the post-intervention was related to mandated reporting; there was no overall change in the number of correct responses to this question after the educational sessions | Significant changes were reported ONLY for awareness of DV resources (1/4 items) | **n/a** | Overall, paediatric health care providers who attended both educational sessions, 46% (16/35) reported that they were routinely screening for the presence of DV at the time of the post-intervention compared with 21% (17/82) prior to the educational sessions (p=.008) | **n/a** | **n/a** |
| **Boursnell and Prosser (2010**) | Nurses self-reported awareness of the policy relating to DV, awareness of their responsibilities to DV increased (sometimes significantly) after they had completed the training programme (Note: 1st post-test n=22). | Nurses’ self-reported knowledge about referrals increased (sometimes significantly) after they had completed the training programme. | Nurses’ self-reported ability to identify children living in DV situations and responding appropriately increased (sometimes significantly) after they had completed the training programme. | **n/a** | The file audit data showed that during May 2007 it was documented in the files of 20% of DV presentations that patients were asked about children. After training, in October 2007, the audit of files indicated that in 66% of DV presentations that patients were asked about children. | **n/a** |
| **CDC (2000)** | **n/a** | **n/a** | **n/a** | **n/a** | IPV screening rates did not increase after implementing on-site victim services (11/14 HPCs did not demonstrate increased screening during phase 2). | **n/a** |
| **Cross and Cerulli (2007)** | No statistical differences were found though there was a trend for the conference group to have higher knowledge scores (8 items). | The groups (intervention vs comparison groups) differed on attitudes behaviours. | The groups (intervention vs comparison groups^[[1]](#endnote-1)^) differed on efficacy behaviours. | The groups (intervention vs comparison groups) differed on practice behaviours (four items). | **n/a** | **n/a** |
| **Haas *et al.* (2011)** | The training did not result in statistically significant changes in the mean levels of knowledge. | The training did not result in statistically significant changes in the mean levels of attitudes & levels of interagency collaboration measures. | **n/a** | CPS workers surveyed after the training^[[2]](#endnote-2)^ had a better relationship with law enforcement and court personnel than a similar sample of CPS workers did before the interagency training class. | **n/a** | **n/a** |
| **Study** | **Knowledge measures** | **Attitudes measures** | **Self-efficacy /competence measures** | **Practice Behaviours/clinical (screening) practice** | **Behaviour change** | **Harm**  **Parental or child anxiety or fear** |
| **Johnson *et al.* (2009)** | Factor analysis was performed on the 18 questions on attitudes/beliefs and self-efficacy by using the varimax rotation for only the baseline data. It was found that all questions could be classified into 5 factors; factors 1 through 5 can be regarded as the measures of “conflict”, “fear of offending”, “self-confidence”, appropriateness” and attitude” respectively. | Yes | Only factor 2 (fear of offending parent) was significantly different from times 1 to 3 (t(67)=2.43, p=0176) indicating that nurses were less fearful after the training. Nurses also reported significant improvement (baseline to follow-up) in several self-efficacy items. | **n/a** | **n/a** | **n/a** |
| **Knapp *et al.* (2006)** | n/a | Significant, favourable changes in attitudes & beliefs between baseline/post-training for 7 statements (11 items in total).  Between baseline/6-month follow-up participants indicated significant, favourable changes in 7(out of 11) attitudes and beliefs questions and in all 7 self-efficacy statements. | Five of the 7 statements regarding self-efficacy showed consistent, significant changes between baseline and post-training. | At the 6-month evaluation only 2 (out of 4) statements showed significant changes; these included the following: ‘In the past year, I have seen a parent/caregiver with an injury and have asked about IPV’ and ‘In the past year, I have seen an abused child and have asked about IPV’. | **n/a** | **n/a** |
| **Lelli (2011)** | There was an increase in preservice teachers’ knowledge pertaining to recognising signs of DV in behaviours of the students they teach. | **n/a** | There was an increase in preservice teachers’ skills pertaining to recognising signs of DV in behaviours of the students they teach | **n/a** | **n/a** | **n/a** |
| **McCauley *et al.* (2003)** | For the 13 questions, physicians and other personnel showed statistically significant improvements for 11 questions. | For the 12 attitude items, statistically significant improvement was present for 10 items in the physician group and 7 questions in the other group. | **n/a** | **n/a** | **n/a** | **n/a** |
| **Study** | **Knowledge measures** | **Attitudes measures** | **Self-efficacy /competence measures** | **Practice Behaviours/clinical (screening) practice** | **Behaviour change** | **Harm**  **Parental or child anxiety or fear** |
| **McColgan et al. (2010**) | Compared to baseline, the 3-month post-intervention assessment revealed significant improvements in perceived knowledge of appropriate IPV screening questions (47.1% vs 100%), referral sources (34.3% vs 82.9%), and the relationship between child abuse and IPV (52.9% vs 97.1%). | Compared to baseline, the 3-month post-intervention assessment revealed significant improvements in 4 (out of 7) attitude questions. | Compared to baseline, the 3-month post-intervention assessment revealed significant improvements in all questions assessing residents’ comfort levels regarding IPV screening. | **n/a** | Significant and sustained improvements in documentation of IPV screening were reported. IPV screening improved from .9% at baseline to 36% at 3 months. IPV screening remained elevated to 33% at 8 months. The IPV counsellor received 107 referrals for IPV during the first 12 months of the intervention; 50 during the first 6 months and 57 during the next 6 months. | **n/a** |
| **Mills & Yoshihama (2002)** | At post-test, participants in the One-Day Programme were significantly more likely to view domestic violence as a social problem; they were significantly less likely to view women as incapable of protecting children and more likely to view women staying in abusive relationships due to their fear of losing custody of the children. | At post-test, participants in the One-Day Programme were significantly less tolerant of DV. | Participants perceived themselves significantly more competent to respond to DV cases following training. | At post-test, participants in the One-Day Programme were significantly more likely to consider assessing whether the mother is being abused as the first tasks of the CSW. | **n/a** | **n/a** |
| **Prather (2003)** | The results indicate a statistically significant difference between groups for five of the subsets of the KAQ: Avoidant Reactions, and its components: lack of recognition, not my role, displacement and sexism, and minimising and victim blaming. | There were no statistically significant between the treatment and the control groups on four of the subsets of the KAQ: Attitude toward oppression, prejudicial attitudes and the subscales of prejudicial attitudes. | **n/a** | Results indicated that students were significantly more aware of appropriate interventions in response to scenario of child abuse and family violence at the termination of the class. Importantly, the employment of interventions that have the possibility to place children and families at risk were also significantly reduced. | **n/a** | **n/a** |
| **Saunders *et al.* (2006)** | **n/a** | **n/a** | **n/a** | **Pre-/post-test evaluation**: workers reported after the training that they would be less likely to refer to couples counselling, and more likely to ask about the emotional and physical impact of abuse, make a safety plan, and ask about access to weapons. Workers most likely to offer a waiver from work requirements reported a higher likelihood of making referrals for a variety of services. | **n/a** | **n/a** |
| **Study** | **Knowledge measures** | **Attitudes measures** | **Self-efficacy /competence measures** | **Practice Behaviours/clinical (screening) practice** | **Behaviour change** | **Harm**  **Parental or child anxiety or fear** |
| **Shefet *et al.* (2007)** | Knowledge of legislation was tested by four true/false questions, pertaining mainly to discrimination between DV cases in which report is mandatory by (Israeli) law and those in which it is not. There was no difference between baseline (1.93) and follow-up score (1.96, t=0.22, p=0.82). | The participants were given a list of potential perceived barriers to interventions in previous cases of DV and were asked to grade them from 1 (did not prevent my intervention at all) to 4 (had strongly prevented my intervention). At follow-up, lack of knowledge and lack of communication skills, as well as unfamiliarity with support systems and psychological difficulties all received significantly lower scores, which indicates an improvement in the physicians’’ attitudes regarding these barriers. | Perceived capability in diagnostic skills, communication skills, knowledge of favourable intervention had increased by 0.29 to 0.6 and were statistically significant (p<.05). | *Frequency of routine screening* of DV has increased (mean score decreased by 0.19, p=0.03); Reported actions – all frequencies of reported actions were increased (all but one increment were of statistical significance); diagnosis and referral. Diagnosis and referral data were also increased (baseline 134 suspicions of DV/74 referrals vs post-test 174 suspicions of DV/116 referrals to relevant agencies). | At baseline, 74 participants reported having encountered altogether 134 cases suspicious of DV in the preceding six months. In 102, the physician further evaluated the suspicion with a detailed history taking and/or physical examination. Seventy-four cases were referred to a relevant agency. At follow-up the same 74 participants reported having suspected 174 cases of DV in the period of six months between the workshop and the follow-up evaluation. In 155 cases evaluation was continued and 116 cases were further referred to relevant agencies. | **n/a** |
| **Young *et al.* (2008)** | Participants indicated at post-test improvements in the hypothesised direction for 4 knowledge items (Questions 1, 3, 6, and 9 - all were statistically significant) | Participants indicated at post-test improvements in the hypothesised direction for 1 attitude item (Question 5 – was statistically significant) | Participants indicated at post-test improvements in the hypothesised direction for 3 skills items (Questions 7, 8, 11- all were statistically significant). | **n/a** | **n/a** | **n/a** |

1. Post-test assessment occurred approximately 6 months following the intervention (i.e. the conference). [↑](#endnote-ref-1)
2. Post-test assessment occurred approximately 6 months following the intervention. [↑](#endnote-ref-2)
