# Supplementary material for: Interventions to Improve the Response of Professionals to Children Exposed to Domestic Violence and Abuse: A Systematic Review
Source: Child Abuse Rev. 2015 Jun 29;26(1):19–39. doi: 10.1002/car.2385 (PMC5363379; doi:10.1002/car.2385)
Supplement: Supplementary file 1 — Supporting info item [file CAR-26-19-s001.zip › CAR-071-14-SI-RESPONDS---Appendix-3b---results-for-RCTs--individual-level-study--March2015_JW.docx]

**Appendix 3b: Results for outcomes in randomised controlled studies (individual-level interventions)**

| **Study** | **Sample** | **Knowledge measures** | **Attitudes measures** | **Self-efficacy /competence measures** | **Clinical (screening) practice** | **Behaviour change** | **Harm**  **Parental or child anxiety or fear** |
| --- | --- | --- | --- | --- | --- | --- | --- |
| **Coonrod *et al.* (2000).** | Maryland, Medical residents entering in 1995 and 1996.  **Experimental group**: n=53/68 randomised  **Control group**: n= 49/68 randomised | **Experimental group**: 73% correct  **Control group**: 56%, p=.002 **^[[1]](#endnote-1)^** | **n/a** | **n/a** | **DV diagnosis**  **Experimental group**: 71%  **Control group**: 52%,  (RR 1.35, 95% CI 0.96 – 1.90) **^[[2]](#endnote-2)^** | **n/a** | **n/a** |
| **Dubowitz *et al.* (2011).** | 18 private practices stratified for size (small, medium, large). Practices ranged from solo to 1 with 32 HPs.  **Experimental group**: 7 practices, median practice size = 5, range = 1-32. 56 participants  **Control group**: 11 practices, median practice size = 3, range = 1-12. 46 participants | SMD 0.28 (95% CI -0.16, 0.72)^[[3]](#endnote-3)^  **18-month follow-up**  SMD 0.24 (95% I -0.20, 0.68)^[[4]](#endnote-4)^  **36-month follow-up**  SMD 0.22 (95% CI -0.38, 0.82)^[[5]](#endnote-5)^ | SMD 0.40 (95% CI -0.04, 0.84)^[[6]](#endnote-6)^  **18-month follow-up**  SMD 0.28 (95%CI -0.16, 0.72)^[[7]](#endnote-7)^  **36-month follow-up**  SMD -0.13 (95%CI -0.73, 0.47)^[[8]](#endnote-8)^ | **Comfort Level^[[9]](#endnote-9)^**  SMD 0.87 (95% CI 0.41, 1.33)  **Perceived Competence^[[10]](#endnote-10)^**  SMD 1.21 (95% CI 0.74, 1.69)^[[11]](#endnote-11)^  **18-month follow-up**  comfort SMD 0.72 (95% CI 0.26, 1.17)^[[12]](#endnote-12)^  competence SMD 1.37 (95%CI 0.88, 1.86)  **36-month follow-up**  comfort SMD 0.79 (95% CI 0.16, 1.41)^[[13]](#endnote-13)^ -  competence SMD 0.59 (95% CI -0.02, 1.20)^[[14]](#endnote-14)^ - | **Practice behaviour**  SMD 0.00 (-0.44, 0.44)^[[15]](#endnote-15)^  **18-month follow-up**  SMD 0.00 (95%CI -0.44, 0.44)^[[16]](#endnote-16)^  **36-month follow-up**  SMD 0.21 (95% CI -0.39, 0.81)^[[17]](#endnote-17)^ | Screened for DV  **Medical records^[[18]](#endnote-18)^**  Pre Post  Intervention: 0.1 23  Control: 0.8 0.8  *β*= 0.18 (95% CI 0.14, 0.22)^[[19]](#endnote-19)^  **Observation of HPs^[[20]](#endnote-20)^**  Pre Post  Intervention: 1 58  Control: 1 2  *β* = 0.43 (95% CI 0.22, 0.63) | **n/a** |
| **Feigelman *et al.* (2011).** | Categorical paediatric & combined medicine-paediatrics residents who provided care in continuity clinics  **Experimental group:** 50 participants  **Control group:** 45 participants | Experimental group improved more than control subjects on IPV psychosocial problem scale **^[[21]](#endnote-21)^** | Experimental group improved more than control subjects on IPV psychosocial problem scale **^[[22]](#endnote-22)^** | Experimental group improved more than control subjects on IPV psychosocial problem scale **^[[23]](#endnote-23)^** |  | **Screened for DV^[[24]](#endnote-24)^**  Pre Post  Intervention: 11/69 255/296  Control: 3/67 29/243  (OR 45.89 (95%CI 27.58 to 76.36) | Parents of children seen by intervention doctors were more satisfied with their child’s doctor compared to those seen by control doctors (PDIS scores 17.4 vs 16.9, p<.01) |

1. Refers to post-intervention knowledge scores measured in 1996; paired analysis, using linear regression to assess treatment while controlling for pre-intervention test score, revealed a significant effect of group (p=.002). [↑](#endnote-ref-1)
2. Refers to self-reported diagnosis of a case of DV sometime between the intervention and the follow-up which occurred 9 to 12 months after the intervention. The intervention group residents were 35% more likely than control residents to diagnose DV. Results, controlling for sex and year, were not different. [↑](#endnote-ref-2)
3. Refers to aggregated knowledge outcome data (for all 4 psychosocial factors); it is difficult to disentangle whether and to what extent the scores reflect changes in knowledge in relation **to DV only**. [↑](#endnote-ref-3)
4. See point 3 [↑](#endnote-ref-4)
5. See point 3 [↑](#endnote-ref-5)
6. Refers to aggregated attitude outcome data (for all 4 psychosocial factors); it is difficult to disentangle whether and to what extent the scores reflect changes in attitudes in relation to DV only. [↑](#endnote-ref-6)
7. See point 6 [↑](#endnote-ref-7)
8. See point 6 [↑](#endnote-ref-8)
9. Refers to aggregated comfort level outcome data (for all 4 psychosocial factors); it is difficult to disentangle whether and to what extent the scores reflect changes in comfort level in relation to DV only. [↑](#endnote-ref-9)
10. Refers to aggregated perceived competence outcome data (for all 4 psychosocial factors); it is difficult to disentangle whether and to what extent the scores reflect changes in perceived confidence in relation to DV only. [↑](#endnote-ref-10)
11. See point 10 [↑](#endnote-ref-11)
12. See point 9 [↑](#endnote-ref-12)
13. See point 9 [↑](#endnote-ref-13)
14. See point 10 [↑](#endnote-ref-14)
15. Refers to aggregated screening practice outcome data (for all 4 psychosocial factors); it is difficult to disentangle whether and to what extent the scores reflect changes in screening practice in relation to DV only. [↑](#endnote-ref-15)
16. See point 15 [↑](#endnote-ref-16)
17. See point 15 [↑](#endnote-ref-17)
18. Medical records of all index children of families participating in the evaluation were reviewed to assess screening for the targeted problems and whether identified problems were addressed. The medical students followed clear guidelines for record abstraction, entering the data on computerised, standardised forms. Questions were resolved with a project paediatrician. [↑](#endnote-ref-18)
19. The *β* values are based on a model that controls for percentage of patients on Medical Assistance in the practice, years that the HP had been in practice, and a random effect of practice. They are interpretable as the difference between study groups with respect to changes in screening percentage points, i.e. *β* =.43 means that the change in screening percentage points for IPV was 0.43 more in the intervention group than in the control group. [↑](#endnote-ref-19)
20. Students observed HPs conducting check-ups, 3 at baseline, and 3 toward the study end. Authors report that the goal was to make the observations as objective as possible; clear guidelines were developed for rating HP actions. For example, “How are you doing?” was not considered a screen for depression. Students coded whether screening occurred for targeted problems and how HPs responded to positive screens. Ratings were entered on a standardized form. [↑](#endnote-ref-20)
21. Mean HPQ scores are presented in a graph (no SDs provided). Scores reflect resident self-assessment on (combined) knowledge, attitudes, comfort, competence and practice regarding screening for DV. This improvement was sustained over 18 months (p=.03, DV) [↑](#endnote-ref-21)
22. Mean PQ scores are presented in a graph (no SDs provided). Scores reflect resident self-assessment on (combined) knowledge, attitudes, comfort, competence and practice regarding screening for DV. This improvement was sustained over 18 months (p=.03, DV) [↑](#endnote-ref-22)
23. Mean PQ scores are presented in a graph (no SDs provided). Scores reflect resident self-assessment on (combined) knowledge, attitudes, comfort, competence and practice regarding screening for DV. This improvement was sustained over 18 months (p=.03, DV) [↑](#endnote-ref-23)
24. Numbers refer to parents screened for IPV before and during the study as documented in the children’s medical charts. [↑](#endnote-ref-24)
