# Supplementary material for: Interventions to Improve the Response of Professionals to Children Exposed to Domestic Violence and Abuse: A Systematic Review
Source: Child Abuse Rev. 2015 Jun 29;26(1):19–39. doi: 10.1002/car.2385 (PMC5363379; doi:10.1002/car.2385)
Supplement: Supplementary file 1 — Supporting info item [file CAR-26-19-s001.zip › CAR-071-14-SI-Responds----Appendix-2----individual-level-study--intervention-characteristics---March2015_JW.docx]

**APPENDIX 2: Characteristics of training programmes (individual-level interventions)**

| **Study** | **Name of intervention** | **Training contents/topics covered** | **Teaching methods** | **Additional resources** | **Delivery format** | **Programme Duration** | **Comparison** |
| --- | --- | --- | --- | --- | --- | --- | --- |
| Berger *et al.* (2002). | n/a | Program was specifically tailored to paediatric health care providers. E.g. focus on the short- and long-term effects of witnessing DV on children.  The content of the sessions was based on the University of Pittsburgh Medical Center Health Systems curriculum on the appropriate health care response to DV (developed at the Magee- Women’s Hospital Violence Center in Pittsburgh). | Didactic session  Follow-up: instruction plus practice/rehearsal and role play | Residents received:  2 articles about DV, a DV Resource pamphlet, a list of local resources. | Group format (including video presentation) | **1^st^:** A **30 minute didactic session** given by one of the authors on 4 separate occasions (Jan 2001).  **Follow-up:** 90-minute teaching session consisting of a 15-minute didactic, 12-minute videotape testimony from DV victims and a 45-minute role-play session. | Baseline scores |
| Boursnell and Prosser (2010) | n/a | Learning outcomes only specified: “By the end of the training participants will be able to identify three key actions in the pathway for suspected DV presentations and locate the pathway for suspected DV presentations within the ED”. | Instruction (how to use the ‘pathway’) & practice discussion (generated by video presentation) | **THE PATHWAY**  A tool specifically developed for use in the ED incorporating existing health service policy and procedures for identifying and responding to DV (e.g. to ask about children and their safety). | Group format incl. video presentation | 45-60 minutes | Baseline scores |
| CDC (2000) | **Pediatric Family Violence Awareness Project (PFVAP**) | AIM of training (Phase 1): to teach HCPs to implement a brief screening protocol of female patients and mothers of paediatric patients aged 0–12 years during routine visits using a recommended screening schedule. | Instruction (training session) | No reference | No reference | 2-hour | baseline |
| Coonrod *et al.* (2000). | n/a | **Experimental group:**  1995: Emphasis on the importance of screening for DV.  1996: A 20-minute programme comprising a nine-minute videotape, “Domestic violence: The bottom line” and a role-play demonstrating interview techniques for detecting DV. | 1995: instruction  1996: instruction + rehearsal/practice | 1996: Selected readings on DV were also provided. | **1995:** 20-minute Video presentation "Domestic Violence: More Prevalent Than You Think,"  **1996**: 9-minute videotape, “Domestic violence: The bottom line” | 1995: 20-minute  1996: 20-minute | **Control groups:** In both years, they attended education sessions on topics unrelated to DV. |
| Cross and Cerulli (2007) | **“Understanding Children Exposed to Community Violence: A Conference for Attorneys Committed to Children,”** | The conference provided information on community violence, local community statistics, evidence-based research on the impact of violence on children, and the rationale and specific strategies for interviewing children as part of the law guardian role. | Presentation (the conference featured four local speakers), Instruction, | Each participant was provided additional reference materials including articles on the topics and copies of the presentations | Large group | single-day professional development training. | Comparison group: non-conference participants recruited via a mail survey |
| **Study** | **Name of training** | **Contents/topics covered** | **Teaching methods** | **Additional resources** | **Delivery format** | **Programme Duration** | **Comparison** |
| Dubowitz *et al.* (2011). | **SEEK (Safe Environment for Every Kid) model of pediatric care** | **Experimental group:**  **HP Training:** The focus was on the significance of targeted problems (parental depression, major stress, substance abuse, and IPV) for children’s health, development and safety, how to briefly assess identified problems, including principles of motivational interviewing. | Instruction (training)  The model recognises the **need for ongoing training**. | The project sent out periodic group newsletters every 9 months. | Small-group training session  The training was conducted by an interdisciplinary team of paediatricians, a SW and a psychologist. | 4-hour  Approx. every 6 months the SEEK group received a ‘booster’ focused on the targeted problems. | HPs in control practices received no special training or SEEK materials; they continued to provide standard paediatric care. |
| Feigelman *et al.* (2011). | **SEEK (Safe Environment for Every Kid) model of pediatric care** | **Experimental group:**  **HP Training**  Training focused on **6 psychosocial risk factors:** parental depression, parental substance abuse, IPV, stress, corporal punishment and food insecurity.  Training was repeated every year. | Instruction (training)  The training was conducted by an interdisciplinary faculty of paediatricians, a SW and a psychologist. | Residents received:  Reference materials, binders with handouts for each targeted problem. | Small-group training session  booster sessions: were interactive and involved discussions of illustrative cases as well as role plays | 8 hour  **booster sessions**: 1-hr were held every 6 months for the interventions groups | Control residents received no special training.  And did not have access to the SEEK training or materials. |
| Haas *et al.* (2011) | n/a | Specific topics covered during the training included: the impact of batterers on adult victims, the public policy principles essential to the legal/court system, an overview of the abuse and neglect process (civil and criminal), the impact of children’s exposure to batterers, assessing the risk across disciplines, planning for safety across disciplines, the roles and responsibilities of other players, bringing the players together when public policy principles conflict, and coordinating community responses.  Participants were expected to begin the process of identifying pathways for overcoming real and perceived barriers and, at the same time, gain an appreciation for the importance of developing interagency collaboration in co-occurrence cases. | Not specified  The curriculum was to be delivered by a multidisciplinary training team—to a multidisciplinary audience—of domestic violence advocates, child protective service workers, law enforcement officers, and court representatives. | n/a | workshop | n/a | Comparison group: sample of CPS workers not exposed to the training |
| Johnson *et al.* (2009) | n/a | The curriculum for IPV screening included 2 components: (1) relevant information; (2) demonstration of the knowledge, skills, and attitudes  Curriculum was based on self-efficacy theory; no other details provided. | Presentation, demonstration, practice opportunities; and feedback | No reference | groups of 2 or more | 30 minute curriculum  20-minute (ONLY for the hospital-produced video about IPV) Total duration of training not reported. | baseline |
| Knapp *et al.* (2006) | **It’s Time to Ask** | **3 modules**: First **module**: basic definitions and concepts regarding IPV in the pediatric health care setting. **Second module**: addressed attitudes, beliefs, and behaviours identified as barriers to screening and intervention. **Third module**: presented a model protocol for use in the paediatric acute care setting. | Instruction (instructional programme) | No reference | No reference | 2-hour | baseline |
| **Study** | **Name of training** | **Contents/topics covered** | **Teaching methods** | **Additional resources** | **Delivery format** | **Programme Duration** | **Comparison** |
| Lelli (2011) | n/a | Professional literature on domestic violence (details provided) | Bibliotherapy | n/a | books | unclear | baseline |
| McCauley *et al.* (2003) | **ASSERT*: A Guide to Child, Elder, Sexual, and Domestic Abuse for Medical Professionals**  * Based on the mnemonic Ask-Sympathize-Safety-Educate-Refer-Treat (ASSERT) | Video content included scripts in four areas of IPV: child abuse, domestic violence, sexual violence, and elder abuse.  It included knowledge and demonstration of clinical skills for IPV identification, prevention, and intervention as defined by the Centers for Disease Control and Prevention (CDC). It addressed physician barriers (identified from published reports) and included material on epidemiology, patient presentations, legal reporting requirements, and recommended treatments. | Instruction, role-play (as it was felt the ‘‘story’’ format engaged the viewer and demonstrated very practical ways to address the difficult issues of IPV in clinical encounters) | No reference | video | One-hour sessions were planned in which the video was introduced briefly to the viewing group by either a social worker or a physician with experience in IPV | baseline |
| McColgan *et al.* (2010) | n/a | **No specific reference made** though it is mentioned that the trainings emphasised the relationship between child abuse and domestic violence. | Talks | See intervention characteristics | No reference  Attendance at the training sessions was expected, but not mandatory. | **Training of the pediatric residents:**  1 hour “grand rounds” presentation to the medical staff; two 1-hour “noon conference” talks on IPV for the residents; and one 25 minute “pre-clinic talk” on IPV screening (by the resident “champion”). | baseline |
| Mills & Yoshihama (2002) | n/a | Both types of training programmes used the Friend, Mills, and colleagues' (1999) curriculum and focused on helping trainees understand the complexities involved in working with families suffering from dual abuses and to develop skills in methods for assessment and intervention. | One-Day Programme: didactic teaching & a role play exercise  Fellow’s programme: six monthly one-day **workshops** | No reference | n/a | One-Day Programme:  Fellow’s programme: six monthly one-day workshops | baseline |
| Prather (2003) | **Child abuse and family violence course (CAFVC)** | The child abuse and family violence course (CAFVC) included specific content and pedagogy in order to directly address the barriers that keep professionals from effectively responding to child abuse and family violence. | Discussion, cooperative learning groups, analysis of case studies, guest speaker didactic instruction as w ell as question and answer. | n/a | Group | Course length was 10-weeks, with class meeting for 2 hours of instruction once a week. | Data from Study 1 participants |
| Saunders *et al.* (2006) | n/a | Covered several key issues: definition & nature of DV, ways victims try to protect themselves and their children, guidelines for interviewing clients, initial inter­ view questions, identifying DV, lethality indicators, helpful interventions, and safety planning tools | Training session – no other details | No reference | n/a | One-day | baseline |
| **Study** | **Name of training** | **Contents/topics covered** | **Teaching methods** | **Additional resources** | **Delivery format** | **Programme Duration** | **Comparison** |
| Shefet *et al.* (2007) | n/a | Each workshop was developed by a national committee of DV experts and included eight scenarios reflecting common DV-related encounters with patients and/or family members and care takers. Each physician encountered two scenarios, and actively viewed, via a one-way mirror, four others. All encounters were audio-visually recorded. Encounters lasted 12 minutes each, after which four minutes were allotted to documentation and comments, and another four minutes for a private, undocumented oral feedback by the actor. At two points during the workshop—halfway through and at the end—the participants assembled in a debriefing room and viewed selected segments of recordings from each encounter. Key points from each of the scenarios (content and/or communication skills) were discussed under the instruction of both a physician and a social worker specialising in DV. | Instruction, practice, feedback  The instructors were pre-trained in a workshop held at MSR, where they experienced all the encounters and discussed the main educational messages of the workshop in general and of each scenario in particular | Complementary material containing articles, relevant resources list and a PowerPoint presentation of the main points, was handed out to participants and/or made available on the MSR intranet website. | workshop | Eight-hour workshop | baseline |
| Young *et al.* (2008) | **“Helping Child Victims of Domestic Violence: Implications for School Personnel”** | Training was developed by a school psychologist using a variety of “best practices” school psychology training programme projects, research articles, and adaptations of existing model training resources. Information for the training on the negative developmental effects of domestic violence and safety planning was adapted from Baker et al. (2004). The sections of the training that pertain to rural domestic violence issues were adapted from the Pennsylvania Coalition Against Domestic Violence (2002) and from Women’s Rural Advocacy Programs (n.d.). | Presentation, Instruction, discussion | The training concluded with information on resources the county domestic violence service providers offer as well as important referral and contact numbers. | Group talk incl. video presentation | The workshop training was approximately an hour and a half in length | baseline |
