# Supplementary material for: Interventions to Improve the Response of Professionals to Children Exposed to Domestic Violence and Abuse: A Systematic Review
Source: Child Abuse Rev. 2015 Jun 29;26(1):19–39. doi: 10.1002/car.2385 (PMC5363379; doi:10.1002/car.2385)
Supplement: Supplementary file 1 — Supporting info item [file CAR-26-19-s001.zip › CAR-071-14-SI-RESPONDS----Appendix-3c---results-for-outcomes-in-system-level-interventions--March2015.docx]

**Appendix 3c: Results for outcomes in system-level interventions^[[1]](#footnote-1)^**

| **Study** | **Sample** | **Knowledge measures** | **Competence measures** | **Clinical (screening) practice** | **Behaviour change** |
| --- | --- | --- | --- | --- | --- |
| **Banks, Landsverk and Wang, (2008b).**  **Main reference** | Five sites  Direct service workers N=578 (total)  Cases reviewed (across sites)  Time1: 616  Time 2: 642  Time3: 562 | Improvements in the following:  - training opportunities in relation to recognising and responding to DV (χ2 = 7.155, df = 1, p = .007).  - agency’s written guidelines for reporting domestic violence to domestic violence service providers (χ2 = 7.024, df = 1, p = .008).  - agency’s written policies that stated when children could remain safely with the nonoffending parent, from 45% at baseline to 68% at follow-up (χ2 = 10.149, df = 1, p = .001).  - close collaboration of their agency with other DV service providers to address co-occurrence (66% at baseline and 83% at follow-up; χ2 = 7.804, df = 1, p = .005)  - sharing of resources with other DV providers (χ2 = 4.905, df = 1, p = .027),. | **n/a** | non-significant changes in :  - proportion of caseworkers agreeing that their agency offered support to battered women in a respectful way without unnecessarily labelling them as being neglectful (scores remained stable over the study period, i.e. approximately 80%)  - proportion of caseworkers agreeing their agencies regularly used a screening and assessment tool at intake and follow-up was not significant (53% at baseline and 63% at follow-up).  - proportion of caseworkers agreeing that their agencies provided voluntary advocacy services for battered women and the proportion of caseworkers who agreed that their agencies referred adult victims of domestic violence to legal services.  - proportion of caseworkers agreeing that their agencies referred battered women to services that would promote self-sufficiency (90% at baseline to 95% at follow-up, with some sites starting at 100%)  - proportion of caseworkers agreeing that they informed adult victims about and referred them to voluntary and community-based services (94% at baseline to 96% at follow-up).  - proportion of caseworkers agreeing that their agencies recorded information in a way that held perpetrators accountable for harm.  - proportion of caseworkers agreeing that that their agencies monitor batterer attendance and compliance with court and programme requirements. | **Evidence of screening** for domestic violence, as found in case files, was highly variable across sites Each demonstration site started and ended quite differently on this measure. Comparing cases across sites opened at Time 1 to those opened at Time 3 showed a significant increase in the proportion of child welfare case files with evidence of active screening for domestic violence (54% at Time 1 and 62% at Time 3; χ2 =7.150, df = 1, p = .007), although the upward trend peaked at Time 2 (77%) and decreased between Time 2 and Time 3. Again, there was great variability among the sites from Time 1 to Time 3.  **Identification of DV cases.** Most sites saw an increase in the proportion of cases in which a history of DV was identified in the child welfare case files. Similar to the active screening measure, the proportion of cases with a history of domestic violence peaked at Time 2 (52%, up from 43% at Time 1) then decreased at Time 3 (47%), but only the increase from Time 1 to Time 2 was significant (χ2 = 10.332, df = 1, p = .001).  **Referrals.** There was a significant increase in the referrals found in child welfare case files for victims of domestic violence from Time 1 to Time 3 (35% to 65%; χ2 = 19.770, df = 1, p = .000), with large site variability in baseline and follow-up values.  **Batterer referrals** A review of evidence from case files showed that there was a significant increase over time across sites. At Time 1, 29% of the case files showed evidence of a batterer referral, followed by a significant increase to 45% at Time 2 (χ2 = 7.550, df = 1, p = .006) and 53% at Time 3 (χ2 = 13.228, df = 1, p = .000). Again, considerable between-site variability was observed. The authors note that the low start values on this measure reflect the need of child welfare agencies to focus on actively engaging and working with batterers at baseline. |
| **Shye *et al.* (2004).** | Clinicians n= 273  Female Patients n = 1925 and n = 1979 for the pre- and post intervention | The ABIS was associated with significantly greater improvement only on knowledge relating to the pros of routine inquiry (*β*= 0.32, p<.0001). | **n/a** | - The ABIS was associated with significantly greater improvement on process of change (b= 0.38, p<.0001). Post intervention scores on perceptions of the medical office social workers as DV experts indicated that improvement was strongly associated with exposure to the social workers’ social change agent role in the ABIS arm (beta for exposure to the ABIS [vs the BIS] = .85, P = .0001 for ABIS exposure and the model).  - At follow-up, 78.8% of ABIS arm clinicians knew about their medical office’s DV response team, compared with only 48.8% of BIS arm clinicians (P = .0001).  - Clinicians in the ABIS arm were much more likely to have learned about the DV guideline from their medical office social worker than were clinicians in the BIS arm. | The odds ratios for the ABIS arm (OR 0.86 95%CI 0.47, 1.55, p=.61) and for the interaction term Time 2 × Study Arm (OR 0.73 95%CI 0.36, 1.47, p=.38) indicate that the ABIS had no greater effect on inquiry rates than the BIS Rather, inquiry rates were a function of patient characteristics and clinician specialty |
| **Wills *et al.* (2008).** | over 700 staff | The sessions effectively increased staff knowledge with identifying and managing child and partner abuse. No other data are provided in the paper. | There is reference to this outcome though actual data are not provided in the paper. ^[[2]](#endnote-1)^ |  | **Referrals^[[3]](#endnote-2)^.** It is reported that the number of notifications from HBDHB ^[[4]](#endnote-3)^to CYFS had increased from 10 per quarter to 70 per quarter^[[5]](#endnote-4)^. CYFS reports indicated that notifications were appropriate and informative, and that interagency relationships were strengthening.  **Screening for partner abuse** is also reported to have been increased in most services, with rates between 6% and 100% recorded during the 2005/06 years, although there was considerable variability in the rate of screening between services. The number of women disclosing abuse was also increased, as was the amount of referral information provided. |

1. Outcomes for two measures, attitudes & parental anxiety and/or fear, are not reported as no study contributed data for these outcomes. [↑](#footnote-ref-1)
2. It is reported that the training component in the study effectively increased staff comfort with identifying and managing child and partner abuse. [↑](#endnote-ref-1)
3. To February 2006, 6176 audits of clinical records for routine questioning for partner abuse had been performed in the services involved in the FVIP. Whether the routine partner abuse question is asked was recorded by staff as a simple +/- tick box in the clinical record. These quarterly audits also assessed the rates of partner abuse disclosure, appropriateness of referrals and that assessment for child abuse was performed when partner abuse was identified. The number of referrals to CYFS for child abuse by HBDHB staff was recorded by CYFS and reported to HBDHB quarterly. [↑](#endnote-ref-2)
4. Hawke’s Bay District Health Board - responsible for the health-care needs of the population [↑](#endnote-ref-3)
5. Documentation audits suggested that the quality of information within clinical records and referrals to CYFS (Department of Child, Youth and Family Services) was increasing, particularly for history/risk assessment and discharge summaries. [↑](#endnote-ref-4)
