# Supplementary material for: Interventions to Improve the Response of Professionals to Children Exposed to Domestic Violence and Abuse: A Systematic Review
Source: Child Abuse Rev. 2015 Jun 29;26(1):19–39. doi: 10.1002/car.2385 (PMC5363379; doi:10.1002/car.2385)
Supplement: Supplementary file 1 — Supporting info item [file CAR-26-19-s001.zip › CAR-071-14-SI-RESPONDS---Appendix--1---Methods--March2015_JW.docx]

**APPENDIX 1**

**Methods**

**Protocol and registration**

The protocol for this review is registered with the PROSPERO database of systematic reviews (<http://www.crd.york.ac.uk/prospero>; registration number CRD42013004672).

**Eligibility criteria**

**Types of studies**

As the aim of the review was to be as inclusive as possible, we did not apply any restrictions in the type of study designs considered for inclusion. Comparisons of interest were no intervention, pre-intervention, wait list control, and alternative interventions.

**Types of participants**

We considered interventions aimed at any professional (e.g. primary care physicians, family doctors, paediatricians, nurses, clinical nurse specialists, practice nurses and health visitors, police officers and teachers) who are in contact with women (aged 15 years and over) and their children who work in any setting including health care, education, criminal justice facilities, and domestic violence agencies.

**Types of interventions**

Any type of intervention or significant change in the national or local policy/practice intended to facilitate and improve professionals’ responses to disclosure of DV with child involvement and improve professional’s response to child maltreatment in the context of domestic violence. Studies could include face-to-face and online single and/or multiagency training or less formal discussion(s) with professionals and could be done in any place, including healthcare services and community setting.

**Types of outcome measures**

The main outcomes of interest were:

1. Professionals’ attitudes, knowledge and competence perception(s) in the assessment and responding to DV disclosure with child involvement;
2. Rates of DV referrals by professionals to other agencies and/or the police.

We also aimed to report all outcome measures reported in the studies that meet our inclusion criteria.

Where possible, outcome data are reported for the short-term (up to one year post-intervention) and long-term (over one year post-intervention).

**Information sources**

Searches were made of the international literature for peer-reviewed and non-peer reviewed studies. There were no language or date restrictions applied to the search strategies used. No trials filters were applied in the searches. The following electronic databases were searched:

| Cochrane Library) | HealthManagement Information Consortium |
| --- | --- |
| MEDLINE | Midwives Information and Resource Index |
| EMBASE | Women Studies International |
| CINAHL | Social Work Abstract |
| ERIC | Family and Society Studies Worldwide |
| ASSIA | Academic Search Complete |
| LILACS | PapersFirst |
| Social Science Citation Index | ProceedingsFirst |
| IBSS | ProQuest Digital dissertations; |
| PsycINFO | National Health and Medical Research Council Clinical Trial Center (NHMRC); |
| British Nursing Index | metaRegister of Controlled Trials |

Every effort was made to identify further studies through web links and websites mentioned in the articles/paper obtained from the primary search. We also examined the reference lists of all acquired papers, and track citations forwards and backwards.

**Selection of studies**

Three review authors (WT, AF and JB) independently screened titles and abstracts in order to exclude studies that were clearly irrelevant. Studies considered eligible by at least one of the reviewers were retrieved in full text. The full texts were then screened by two members of the review team (WT and AF or JB) to determine study eligibility based on the inclusion criteria. Any disagreements about eligibility were resolved by another review author (GF). Reasons for exclusion were documented for each study that is retrieved in full text. The overall search and screening process is illustrated in a flow-diagram (Figure 1). Authors were not blinded to the name(s) of the study author(s), their institution(s) or publication sources at any stage of the review.

**Data extraction and management**

Data were extracted by one review authors (WT) using a specially designed data extraction form with a second review author (GF) performing a validation check (on at least 10% of the included studies). Study authors were contacted for clarification and/or missing information. Data were excluded until further clarification was made available by the study’s author(s) and/or if the study is unobtainable.

For each trial the following data were recorded:

- Setting of the professionals targeted by the intervention;
- Professional’s information (e.g. title, speciality, work setting);
- Parent’s and children’s information - age, sex, symptoms and duration (if their outcomes are measured in the study);
- Information (from parents and their children) on reasons for seeking professional help, diagnosis verification.
- Intervention - the type of intervention and procedural information.
- Study design - method of allocation, sample size, blinding of participants and outcomes, inclusion and exclusion criteria, proportion of follow-up losses.
- Outcomes reported in the study;
- Additional information - country of origin of the study, language of publication, date of publication and source of article (e.g. a database).

**Assessment of risk of bias in included studies**

Two reviewers (WT and GF) independently assessed the risk of bias for each included study, using the ‘Risk of bias’ tool described in the Cochrane Handbook for Systematic Reviews of Interventions ([Higgins and Green 2011](file:///G:\GID%20protocol%20files\Behavioural%20and%20cognitive%20behavioural%20psychotherapies%20for%20Gender%20Identity%20Disorder%20%28GID%29%20in%20childhood-v2.htm#REF-Higgins-2008)) and additional criteria developed by the Cochrane EPOC Group ([EPOC 2009](file:///G:\GID%20protocol%20files\Behavioural%20and%20cognitive%20behavioural%20psychotherapies%20for%20Gender%20Identity%20Disorder%20%28GID%29%20in%20childhood-v2.htm#REF-EPOC-2009)).

For RCTs and CBAs we assessed risk of bias associated with the following six domains:

1. sequence generation;
2. allocation concealment;
3. blinding of outcome assessors;
4. incomplete outcome data;
5. selective outcome reporting; and
6. other sources of bias ([Higgins and Green 2011](file:///G:\GID%20protocol%20files\Behavioural%20and%20cognitive%20behavioural%20psychotherapies%20for%20Gender%20Identity%20Disorder%20%28GID%29%20in%20childhood-v2.htm#REF-Higgins-2008)).

As advised by the Cochrane EPOC group ([EPOC 2009](file:///G:\GID%20protocol%20files\Behavioural%20and%20cognitive%20behavioural%20psychotherapies%20for%20Gender%20Identity%20Disorder%20%28GID%29%20in%20childhood-v2.htm#REF-EPOC-2009)), we also included three additional domains that address design-specific threats to validity:

1. imbalance of outcome measures at baseline
2. similarity of baseline characteristics between control and intervention group, and
3. protection against contamination

For ITS or B/A studies, we assessed risk of bias associated with the following seven domains:

1. intervention independent of other changes;
2. shape of intervention effect pre-specified;
3. intervention unlikely to affect data collection;
4. blinding of outcome assessors to intervention allocation;
5. incomplete outcome data;
6. selective outcome reporting; and
7. other sources of bias (EPOC 2009).

The criteria specified in the Cochrane Handbook for Systematic Reviews of Interventions ([Higgins and Green 2011](file:///G:\GID%20protocol%20files\Behavioural%20and%20cognitive%20behavioural%20psychotherapies%20for%20Gender%20Identity%20Disorder%20%28GID%29%20in%20childhood-v2.htm#REF-Higgins-2008)) and the guidance offered by Cochrane EPOC Group ([EPOC 2009](file:///G:\GID%20protocol%20files\Behavioural%20and%20cognitive%20behavioural%20psychotherapies%20for%20Gender%20Identity%20Disorder%20%28GID%29%20in%20childhood-v2.htm#REF-EPOC-2009)) guided our judgement as to whether a study has a low ('Yes' judgement), high ('No' judgement), or uncertain ('Unclear' judgement) risk of bias for each domain. For each included study, we aimed to report our assessment of risk of bias for each domain together with a descriptive summary of the information that influenced our judgment.

**Measures of treatment effect**

Where dichotomous (binary) data are presented, the odds ratio (OR) with a 95% confidence interval was used to summarise results for each outcome in each trial ([Lipsey and Wilson, 2001](file:///G:\\GID%20protocol%20files\\Behavioural%20and%20cognitive%20behavioural%20psychotherapies%20for%20Gender%20Identity%20Disorder%20%28GID%29%20in%20childhood-v2.htm#REF-Lipsey-2001)). Where results were reported in short ordinal scales, the methods of Whitehead and Jones were used to produce a single odds ratio from each trial ([Whitehead and Jones, 1994](file:///G:\GID%20protocol%20files\Behavioural%20and%20cognitive%20behavioural%20psychotherapies%20for%20Gender%20Identity%20Disorder%20%28GID%29%20in%20childhood-v2.htm#REF-Whitehead-1994)). If sufficient detail was not provided we analysed such scales as continuous data after investigating skew and appropriateness. Where the outcomes measures arose from ordinal rating scales and the rating scales used have a reasonably large number of categories (more than 10) the data was treated as continuous variables arising from a normal distribution. The same approach with ordinal scales with a moderate number of categories was followed. The mean difference (MD) was generated for ordinal data where the data was provided as a mean and standard deviation.

Continuous data were analysed if means and standard deviations were presented in the study papers, were made available by primary investigators or were calculable from the available data. If continuous outcomes were measured identically across studies, the mean difference (MD) with 95% CI was calculated. If the same continuous outcome was measured differently across studies, standardised mean differences (SMD) with 95% CI was compared across studies ([Higgins and Green, 2011](file:///C:\Users\sozjef\AppData\Roaming\Cyrusoft\Mulberry\Temporary%20Files\View%20Attachments\Higgins%202011)) Where necessary, we aimed to use formulas to convert F ratios, t-values and Chi^2^ values into SMDs ([Lipsey and Wilson, 2001](file:///C:\\Users\\sozjef\\AppData\\Roaming\\Cyrusoft\\Mulberry\\Temporary%20Files\\View%20Attachments\\Lipsey%202001)) using Hedges *g* to correct for small sample bias. Inverse variance methods would have been used to pool SMDs, so that each effect size is weighed by the inverse variance in an overall estimate of effect size.

Continuous data that are skewed are reported in a separate table; in such cases, we did not calculate treatment effect sizes to minimise the risk of applying parametric statistics to data that depart significantly from a normal distribution. We define skewedness as occurring when, for a scale or measure with positive values and a minimum value of zero, the mean is less than twice the standard deviation (Deeks, 1997a, b). If studies were considered to have more than one eligible outcome for a forest plot, we would have selected a single outcome based on expert advice, principally the most commonly used with the highest validity.

**Assessment of heterogeneity**

Heterogeneity among included studies was examined through a) the use of the chi^2^ test, where a low p-value indicates heterogeneity of treatment effects, b) the I^2^ statistic, to determine the percentage of variability that is due to heterogeneity rather than sampling error or chance, and c) by comparing the results of fixed and random effects models ([Higgins and Thompson, 2002](file:///G:\GID%20protocol%20files\Behavioural%20and%20cognitive%20behavioural%20psychotherapies%20for%20Gender%20Identity%20Disorder%20%28GID%29%20in%20childhood-v2.htm#REF-Higgins-2002)). Possible sources of heterogeneity were investigated; if there was evidence of heterogeneity (i.e. the statistical test of heterogeneity is statistically significant and I^2^ > 50%) ([Higgins and Thompson, 2002](file:///\\5LESLES36.SOUTHWARKPCT.NHS.UK\VOL1\GUUSERS\WalshJu\Downloads\CAR-071-14-main%20txt-response%20to%20Ed%20comments%20--%20March2015.docx)) and sensitivity analyses, where data permit, were conducted.

**Data synthesis**

Studies were grouped according to study design. Results for RCTs, CCTs and CBAs outcome data are reported separately and were pooled only if sufficient clinical and methodological homogeneity exists between studies. Data synthesis was conducted using RevMan 5. For Interrupted time series (ITS) studies we aimed to calculate relative and absolute mean difference in before and after values. When possible, we aimed to use time series regression analyses to calculate mean change in level and mean change in slope. Where possible meta-analysis was conducted on the combined results of homogeneous studies using a random effects model.

If due to substantial and unreconciable heterogeneity, meta-analysis was not feasible we present a narrative synthesis of the studies based on study design, quality, the size, direction and significance of observed effects and consistency of findings (number of studies using an approach reporting a similar sized same effect out of the number of studies using the approach reporting no effect). Based on this, a conservative appraisal of the merit of the intervention has been provided. It should be acknowledged that this was not the preferred course of action as such synthesis would not produce any clear (or robust) recommendation for practice and policy. It constitutes though a transparent appraisal of systematically gathered studies which might prompt further rigorous studies in this field.

**Subgroup analysis and investigation of heterogeneity**

Methodological and clinical heterogeneity would be explored in any possible meta-analysis. Based on the availability of data from the included studies, the following subgroup analyses to explore the influence of important factors on the results would be performed:

- Setting in which the intervention was taking place, duration of the intervention and delivery method (individual vs. group);
- variations in comparison condition (if found);
- duration of follow-up;
- study quality (comparison of high and low quality studies)

**Sensitivity analysis**

In order to determine how robust and consistent the results are, sensitivity analyses would be conducted based upon study design (RCT vs. other), level of attrition and risk of bias in study (high, moderate, low - according to the Effective Practice and Organisation of Care Group’s (EPOC) quality checklists).

Comparison data for each design are reported separately. Consideration was given on outcome heterogeneity associated with study design.

**Appendix 1 --Figure 1. PRISMA Flow diagram of screened and included papers**
